# Examining the Impact of Side-Chain Chirality on Conformation of a Helical Poly(3‑(*S*‑1-ethylhexyl)esterfuran)

**DOI:** 10.1021/acs.macromol.5c01499

**Published:** 2025-11-15

**Authors:** Dhruv Sharma, Manami Kawakami, Megan Rice, Erin Smith, Soren Westrey, Yuyang Shang, Claire Gist, Leticia Maria Pequeno Madureira, Karl H. G. Schulz, Anthony J. Varni, Isabella M. Stranick, Roberto R. Gil, Stephanie Tristram-Nagle, Linda Peteanu, Tomasz Kowalewski, Kevin J. T. Noonan

**Affiliations:** † Department of Chemistry, 6612 Carnegie Mellon University, 4400 Fifth Avenue, Pittsburgh, Pennsylvania 15213, United States; ‡ Physics Department, Carnegie Mellon University, Pittsburgh, Pennsylvania 15213-2617, United States

## Abstract

A key challenge with synthetic chiral helical polymers is the precise determination of their structure, particularly their pitch and handedness. In past work, we demonstrated that poly­(3-hexylesterfuran) (P3HEF) adopts a compact helical conformation (pitch ∼3.4 Å), driven by the *syn* conformational preference for regioregular, α-linked furan-3-carboxylates. Chiral side chains (either *R* or *S*) were attached to the furan monomer to synthesize poly­(3-(1-ethylhexyl)­esterfurans) (*R*- or *S*-P3­(1EH)­EF) with excess helix sense, but the branched alkyl group clearly impacted the folding behavior of these polymers. Here, through combined experimental and computational analyses, we assigned helix sense in these ester-functionalized polyfurans: where the *S* configuration for the side chain results in a left-handed helix bias, while the opposite *R* enantiomer results in a right-handed helix bias. While helix handedness can be biased by the attachment of the chiral side chain, the ethyl branch disrupts the formation of well-ordered helices when compared to the P3HEF analog. Solid-state characterization of *S*-P3­(1EH)­EF revealed isotropic grazing-incidence wide-angle X-ray scattering (GIWAXS) patterns, in contrast to the anisotropic edge-on orientation observed for P3HEF films. Thermal analysis with powder X-ray diffraction showed that while P3HEF remains stable up to 350 °C, the *S*-P3­(1EH)­EF melts near 150 °C in the solid state. Additionally, the chiral conformation of the *S*-P3­(1EH)­EF polymer is lost upon heating in THF solution above 30 °C, as evidenced by temperature-dependent circular dichroism (CD) studies. The results demonstrate that while the 1-ethylhexyl chiral side chains can bias helix sense, they also partially disrupt the formation and stability of a compact helical structure for ester-functionalized polyfurans.

## Graphical abstract

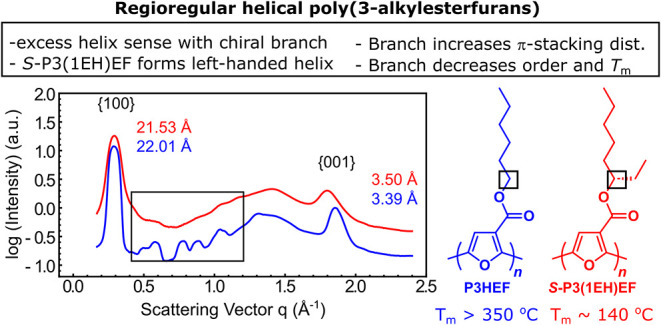

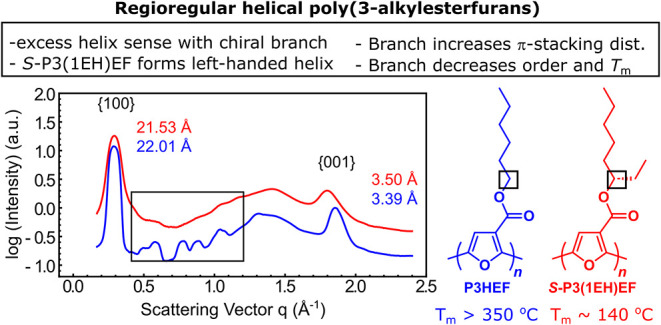

## Introduction

Chiral conjugated polymers^1–6^ (CCP) are a class of optically active and electroactive macromolecules with potential properties ranging from chiral recognition, to circularly polarized light (CPL) emission,^7–10^ to chiral induced spin selectivity.^11,12^ CCPs are often prepared by attaching solubilizing side chains with point chirality to a conjugated polymer backbone. Chiroptical properties in CCPs generally arise through one of two mechanisms: either via the self-assembly of polymers into chiral aggregates (typically driven by π–π stacking in poor solvents or thin films), or through the adoption of a helical conformation by the polymer chain itself, resulting in an excess of one helix sense (Figure 1A).^13^

**Figure 1:**
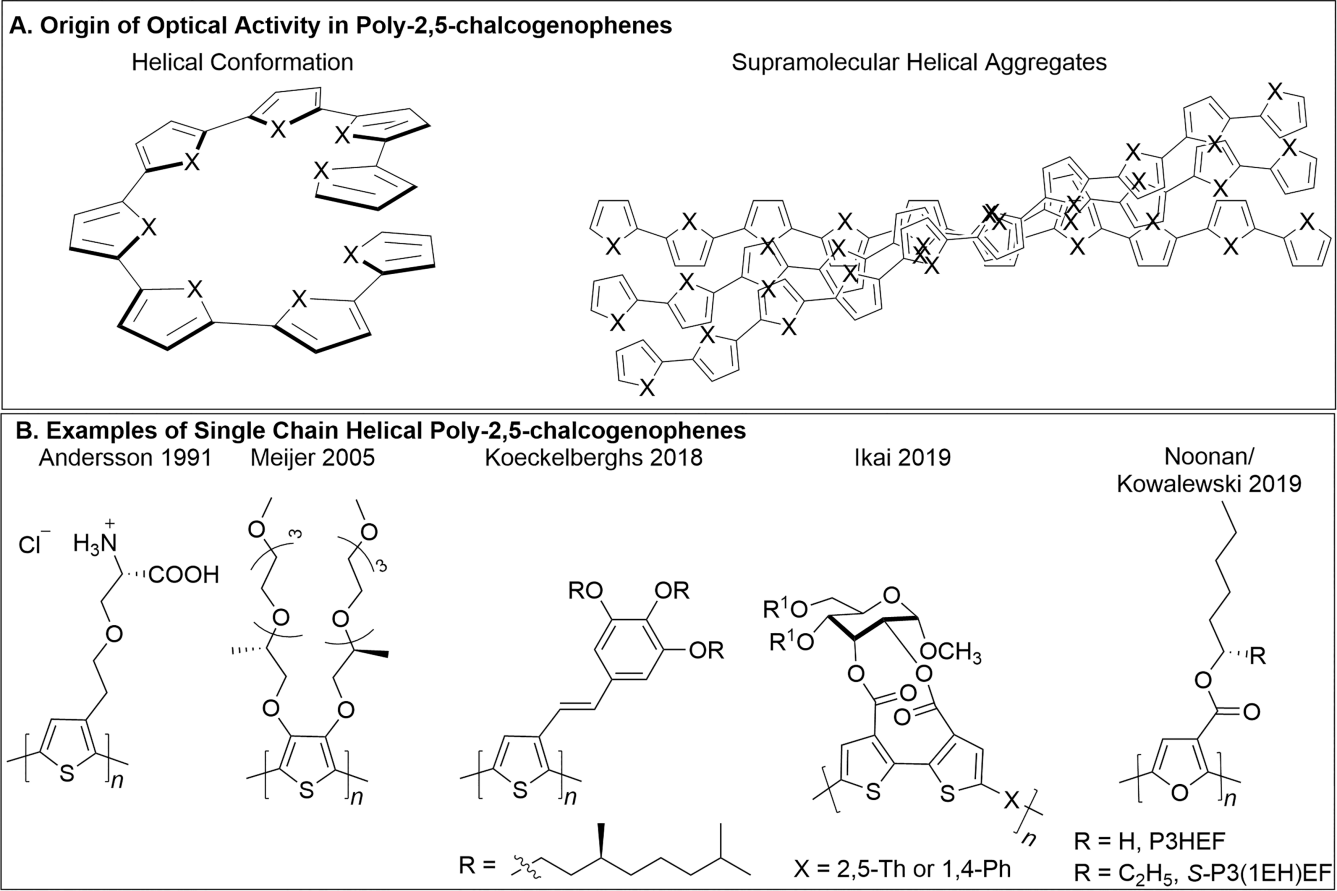
(A) Origin of optical activity in polychalcogenophenes (adapted from reference 13). (B) Some examples of poly-2,5-chalcogenophenes which adopt a single-chain helical conformation.

Thiophene is a common building block in CCPs since chiral side chains can be readily installed on the ring prior to polymerization.^1^ Furthermore, under suitable conditions, chain-growth cross-coupling polymerization^14–18^ enables the synthesis of well-defined poly­(3-substituted thiophenes) with pendant chiral side groups,^19–23^ offering precise control over molecular weight and end-group functionality. This approach also enables access to other polymer architectures such as block copolymers.^24–26^ Despite the broad range of reported chiral polythiophenes (PTs),^1^ only a few examples of single-chain PT helices have been described (Figure 1B),^21,27–34^ and the expansion to other helical polychalcogenophenes^35^ based on furan, selenophene, and tellurophene are limited. Varying the heteroatom in the repeat unit (O, S, Se, Te) will have an impact on the size, shape, stability, and electronic properties of the resulting helix and should offer insight into the factors governing helix formation in polychalogenophene CCPs.

In our work, we recently discovered that poly­(3-hexylesterfuran) P3HEF spontaneously forms a helical structure,^36^ as noncovalent interactions drive adjacent furan-3-carboxylates to adopt a *syn* conformation. The regioregular P3HEF is unique, as it does not unfold upon heating in solution (up to 80 °C) and no melt transition is observed in the solid state (up to 350 °C). It can be made chiral by attachment of enantiopure chiral alcohols as side chains, and in our initial report, we observed that a *S*-1-ethylhexyl ester group on the polymer (*S*-P3­(1EH)­EF in Figure 1) induced excess helix sense, as determined by circular dichroism (CD) studies conducted in CHCl_3_/MeOH mixtures.^36^ We also noted that the branched side chain significantly disrupted the helical conformation in CHCl_3_,^36^ which prompted us to examine how the chiral side chain dictates helix sense and molecular packing.

Herein, computation, solid-state, and solution-phase studies were used to address two key questions with the chiral P3­(1EH)­EF: what is the handedness of the helical polymer and to what extent is the helical geometry of the polyfuran disrupted with the chiral side chain as compared to the achiral analog with a linear side chain (poly-(3-hexylesterfuran) or P3HEF). Computational studies using density functional theory (DFT) enabled tentative assignment of the helix handedness by comparison with experimental data. X-ray scattering studies clearly demonstrated that the branched side chain in *S*-P3­(1EH)­EF frustrates adoption of a compact helical structure as compared to the achiral analog. While P3HEF displays no melt transition when heated to just below its decomposition temperature, *S*-P3­(1EH)­EF melts near 150 °C. In THF, variable-temperature CD studies revealed the helix can be partially melted just above room temperature, with near complete loss of the Cotton effect above 30 °C. Finally, solid-state CD studies also confirmed how sensitive the chiroptical response is to solvent environment and preaggregation, as the chiroptical response changes dramatically upon drop-casting from either CHCl_3_, THF, or *n*-octane.

## Results and Discussion

### Helix Sense

Expanding on prior work,^36,37^ we computed left (*M*) and right (*P*) helices for regioregular ester-functionalized polyfurans to examine whether the predicted CD spectra would match with experimental data (Figure 2). Geometry optimizations of the left- and right-handed helices (*M* and *P*) of a poly­(3-methylesterfuran) 13-mer (P3MEF) were performed using density functional theory (DFT) calculations with the dispersion-corrected B3LYP-D3­(BJ) functional and the 6–31G­(d,p) basis set. The 13-mer serves as a reasonable representation of the helical polymer, consisting of two helix turns (Figure 2A1,A2). The methyl ester side group was used to decrease computational cost, and the computed total energies for both structures were within error, as expected.

**Figure 2:**
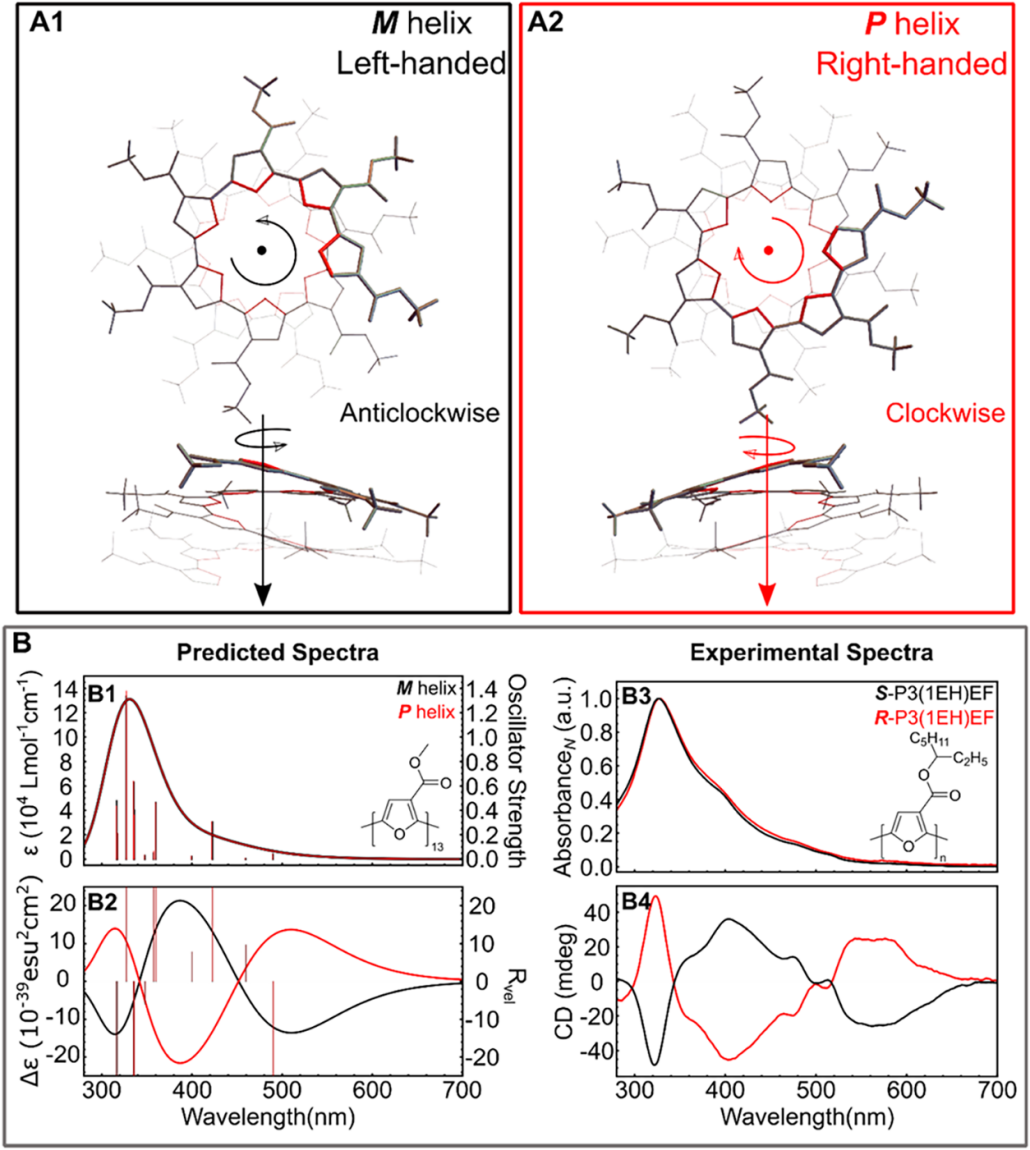
(A1, A2) DFT-optimized left- and right-handed helical structures of P3MEF using B3LYP-D3­(BJ) functional and a 6–31G­(d,p) basis set. (B1, B2) TDDFT-predicted absorption and CD spectra (CAM-B3LYP/6–31G­(d,p)) for regioregular P3MEF in *M* (black) and *P* (red) configurations. The standard deviation (σ) for the width of the bands is 0.333 eV. (B3, B4) Normalized absorption and CD spectra of *S*-P3­(1EH)­EF (*M_n_* = 11.7 kg/mol) and *R*-P3­(1EH)­EF (*M_n_* = 10.2 kg/mol) in a 40:60 CHCl_3_/CH_3_OH mixture (0.02 mg/mL solutions). The absorption and CD spectra are reproduced from reference 36.

Time-dependent density functional theory calculations (TDDFT CAM-B3LYP/6–31G­(d,p)) were then used to predict the absorption and CD spectra which are shown in Figure 2B (B1 and B2), and these were compared to the original spectra collected for the *S*- and *R*-P3­(1EH)­EF in 4:6 CHCl_3_/MeOH (B3 and B4). Helical P3AEFs have a very distinct absorption spectrum with a λ_max_ ∼ 330 nm and a long tail to near 600 nm. This signature is a function of the weakly allowed highest occupied molecular orbital–lowest unoccupied molecular orbital (HOMO–LUMO) (π–π*) transition with all of the furan-3-carboxylates in a *syn* conformation.^36^ This predicted absorption spectrum changes dramatically if the furan repeat units are all in an *anti* conformation, with a large oscillator strength for the π–π* transition (Figure S33). Natural transition orbital (NTO) representations with their associated transition dipole moments were computed for the all-*syn* and all-*anti* conformations of the polymer using TDDFT (CAM-B3LYP/6–31G­(d,p)). These calculations highlight how the π orbitals of the core furan-3-carboxylate repeat units are involved in the first excited state (Figures S34 and S37), and how low the oscillator strength is when the polymer adopts the helical conformation and the transition dipole moment is along the helix axis (*f* = 0.04). The absorption spectra for the synthesized *S*- and *R*-P3­(1EH)­EF are a good match to the predicted spectra, and the computed CD spectra are also in good agreement with the experimental data (Figure 2B). These results suggest a tentative assignment can be made where the *S*-P3­(1EH)­EF adopts a left-handed helix (*M*) and *R*-P3­(1EH)­EF adopts a right-handed helix (*P)*.

To gain further insight into the origin of helix bias arising from the side chain, computational studies were performed with the chiral *S*- or *R*-1-ethylhexyl side group explicitly included. Geometry optimizations were conducted on α-linked oligomers of furan-3-carboxylates ranging from 6 to 14 repeat units (Figure 3). These optimizations were carried out using the GFN2-xTB method,^38–40^ a semiempirical tight-binding approach designed for rapid calculations and accurate treatment of noncovalent interactions in larger molecular systems.

**Figure 3:**
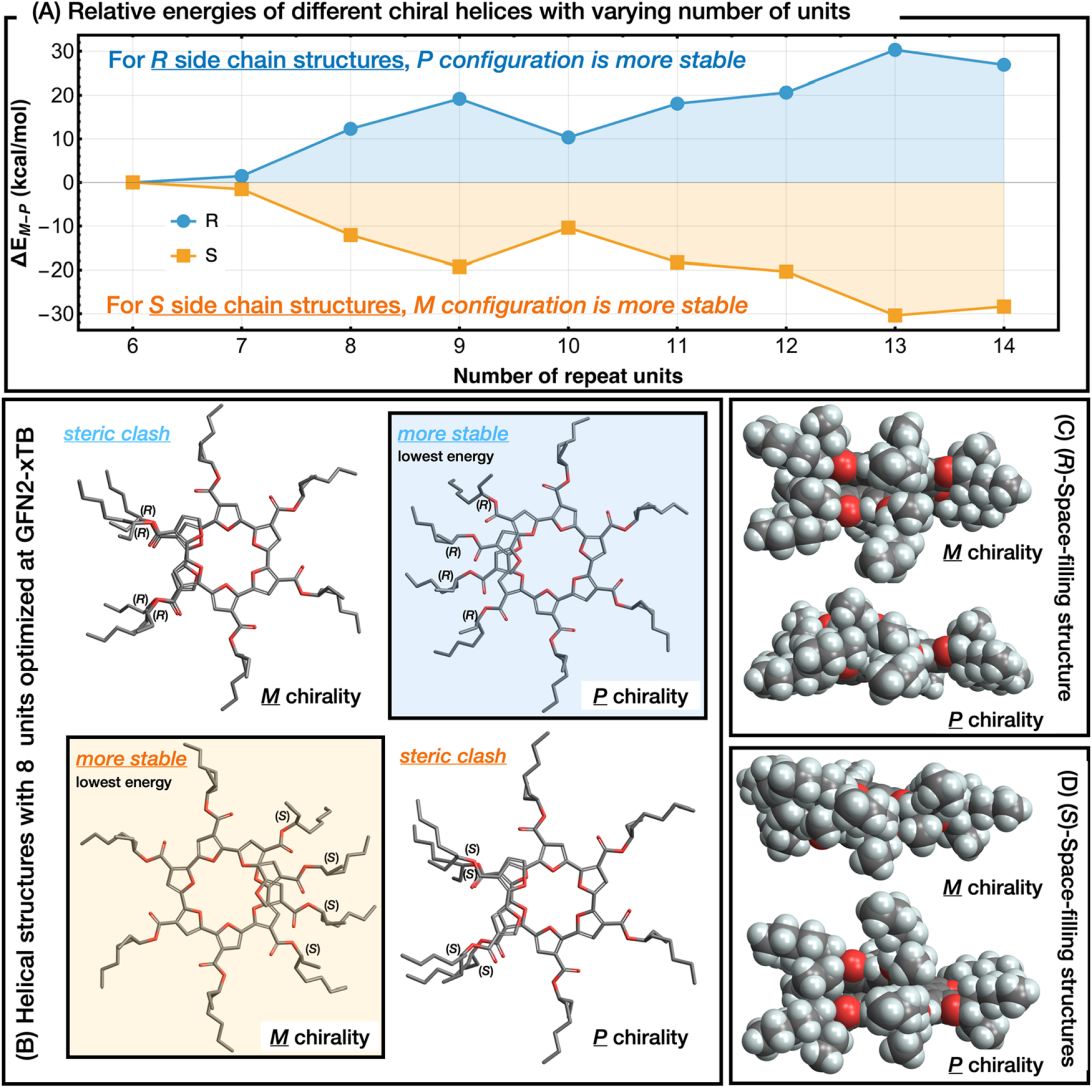
Chirality-directed helix-sense bias and representative structures. (A) Δ*E*(*M*–*P*) vs repeat unit number (*n* = 6–14) for *R* (blue) and *S* (orange) side chains, showing that *R* side chains increasingly favor *P* helices and *S* side chains favor *M* helices *n* ≥ 8. (B) GFN2-xTB optimized conformers of the eight-unit oligomer: top, *R* series; bottom, *S* series; left, *M* helix; right, *P* helix. (C, D) Space-filling models of the minimum-energy *M* and *P* helices for (C) *R*-substituted and (D) *S*-substituted series, illustrating how side-chain stereochemistry propagates long-range helical preference.

Minimal energetic differences were observed for oligomers with chain lengths of 6 or 7 repeat units (Figure 3A). At these chain lengths, the helical backbone is too short for side chains to interact meaningfully, and the energy difference (Δ*E*) between the *M* and *P* helices remains within the range of thermal fluctuations. However, when the oligomer length increases to 8 repeat units or more, the helical pitch brings side chains into proximity. For the *R*-configured side chain, the *P* helix becomes energetically favored, with an energy difference of up to ∼30 kcal mol^–1^ for the 13-mer. Conversely, the *S*-configured side chain favors the *M* helix by a similar magnitude for the same oligomer length. In these matched configurations (*R*–*P* or *S*–*M*), the alkyl side chains interdigitate along the helical groove in a complementary fashion (Figure 3B), maximizing dispersion interactions and minimizing steric clashes. In contrast, mismatched pairs (*R*–M or *S*–P) show significant steric repulsion, as side chains on neighboring turns overlap. This is exemplified by the high-energy conformers in Figure 3B (upper left and lower right), where methylene groups intrude into one another’s space, resulting in a substantial energetic penalty.

The space-filling renderings in panels C and D of Figure 3 further underscore how stereochemistry dictates three-dimensional packing. In the lowest-energy *R*–*P* structure (Figure 3C, bottom), the extended hexyl arms fold neatly along the helix surface, creating a uniform, contiguous cylinder of hydrophobic contact that both shields the backbone and distributes steric bulk evenly. In contrast, the mismatched *R*–*M* model (Figure 3C, top) shows pronounced protrusions of the butyl termini into adjacent turns, forcing local backbone distortion to relieve clashes. A similar contrast is observed for *S*-substituted oligomers (Figure 3D): the stabilized *S*–*M* helix adopts a regular, staggered side-chain array, whereas the disfavored *S*–*P* helix exhibits localized kinks and widened helical pitch to accommodate steric crowding. Together, these structural snapshots reveal that helix sense emerges from a delicate balance between backbone torsional strain and side-chain packing efficiency, with steric complementarity acting as the primary driver of long-range chiral amplification.

### Disorder from the Branched Side Chain

Once the helix sense of the P3­(1EH)­EF was established, P3HEF and *S*-P3­(1EH)­EF were resynthesized to evaluate how the ethyl branch impacts order. For the experimental work, we focused on the *S*-P3­(1EH)­EF since *S*-3-octanol is commercially available. P3HEF was prepared as previously reported,^36^ and *S*-P3­(1EH)­EF was prepared using the commercially available dichloro­[1,3-bis­(2,6-di-3-pentylphenyl)­imidazol-2-ylidene]­(3-chloropyridyl)­palladium­(II) (Pd-PEPPSI-IPent). We have noted that this catalyst affords regioregular ester-functionalized polychalcogenophenes with relatively high molecular weights in Suzuki-Miyaura cross-coupling polymerization.^37,41^ Two samples of the chiral polymer were synthesized (14.9 and 19.4 kg/mol) and one sample of the achiral P3HEF (8.2 kg/mol).

As has been reported,^36^ a sharp and well-defined ^1^H NMR spectrum is observed for *S*-P3­(1EH)­EF in CDCl_3_ (Figure 4), since the polymer is partially unfolded in that solvent. This contrasts with the broadened signals observed in the ^1^H NMR spectrum for P3HEF in CDCl_3_ (Figure S6).^36,37^ The *S*-P3­(1EH)­EF is regioregular, as evidenced by the single aromatic signal (*H*_A_ at 7.78 ppm) and a single methine group at 5.06 ppm (*H*_B_) observed in the ^1^H NMR spectrum (Figure 4).

**Figure 4:**
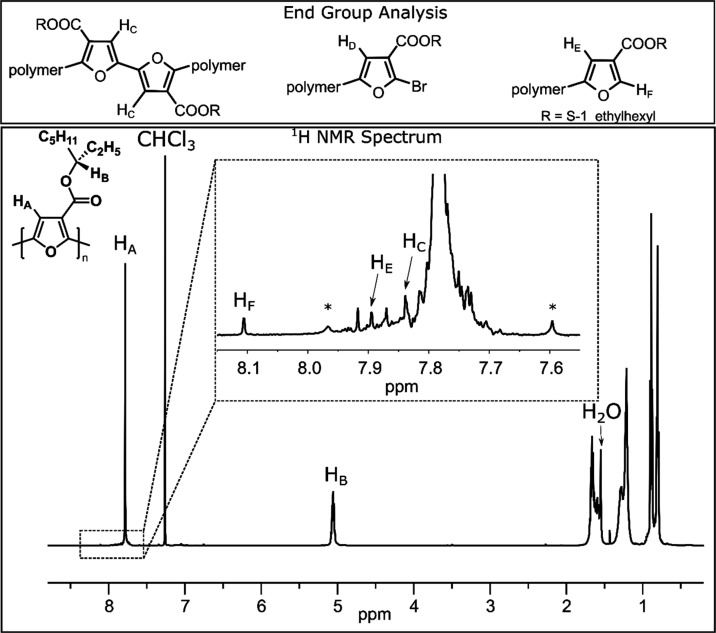
^1^H NMR spectrum (500 MHz, 25 °C) of *S*-P3­(1EH)­EF (*M_n_* = 14.9 kg/mol) collected in CDCl_3_. Inset corresponds to an expansion of the aromatic region at higher intensity to visualize the minor signals. Assignments for the TT defect and H-terminated end group are labeled. The signals labeled with a * correspond to the ^13^C satellites for the polymer main chain.

Several low-intensity signals were observed in the aromatic region of the NMR spectrum (Figure 4), which likely correspond to end groups and minor structural defects. Some of these signals were assigned with the aid of two-dimensional NMR techniques, including COSY and HMBC (Figures S3 and S4). Because a metal dihalide precatalyst was used to initiate polymerization, a tail-to-tail (TT) defect is expected within the polymer chain.^42–44^ The signal for the TT defect was tentatively assigned to the peak at 7.84 ppm (*H*_C_), based on correlations observed in the HMBC spectrum (Figure S4). Two aromatic signals arise for the H-terminated end group, which appear at 7.89 ppm (*H*_E_) and 8.11 ppm (*H*_F_), with clear cross-peaks in the COSY spectrum (Figure S3). However, the proton corresponding to the anticipated Br-terminated end group (*H*_D_) could not be confidently assigned, as the expected correlation was not clearly observable in the HMBC spectrum. Additional minor aromatic signals may arise from other end groups,^45^ or from repeat units adjacent to the end groups and the TT defect.

### Solid-State Morphology

Increased disorder in the molecular packing of the chiral polymer, relative to its achiral analog, was evident from both powder X-ray diffraction (XRD) and grazing-incidence wide-angle X-ray scattering (GIWAXS) patterns (Figure 5). Powders of P3HEF and *S*-P3­(1EH)­EF were obtained by slow evaporation from THF solutions, while ultrathin films of the same materials were prepared by drop-casting from CHCl_3_ onto silicon wafers.

**Figure 5:**
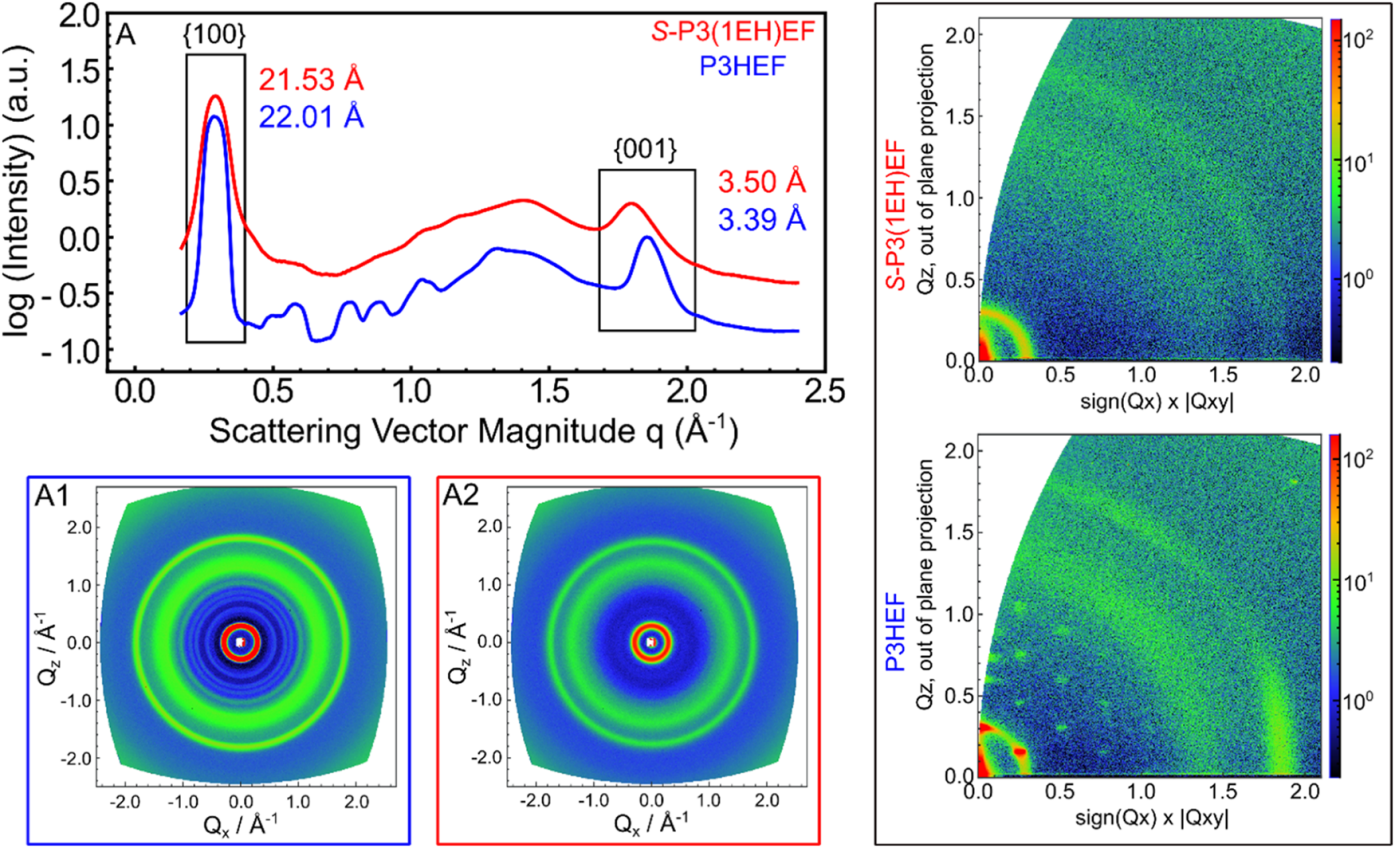
(A) Azimuthally averaged powder diffraction patterns obtained for P3HEF (*M*_*n*_ = 8.2 kg/mol, *Đ* = 1.28) (blue and A1) and *S*-P3­(1EH)­EF (*M*_*n*_ = 14.9 kg/mol, *Đ* = 1.36) (red and A2). Data were collected at 25 °C. Samples were prepared by slow evaporation from 2 mg/mL THF solutions. Right - GIWAXS patterns collected for thin films of P3HEF (*M*_*n*_ = 8.2 kg/mol, *Đ* = 1.28) and *S*-P3­(1EH)­EF (*M*_*n*_ = 14.9 kg/mol, *Đ* = 1.36) cast onto silicon wafers from 5 mg/mL solutions in CHCl_3_.

The powder patterns for P3HEF (blue in Figure 5) suggest a high degree of order, as evidenced by the well-defined pattern in the 0.5 to 1.5 Å^–1^ range of scattering vector magnitude (*q* = 2π/*d*, where *d* is the interplanar spacing) which is attributed to side-chain packing (consistent with prior work).^36^ This region is much less defined for the chiral analog (red in Figure 5). Distinct hexagonal packing of π-stacked helical assemblies were noted for P3HEF in GIWAXS patterns,^36^ with the polymer oriented edge-on with respect to the substrate (Figure 5). It should be noted that while the orientation is predominantly edge-on, a fraction of the population appears off-axis, indicating an intermediate orientation between face-on and edge-on relative to the substrate. The edge-on population (π-stacking close to 0°) accounts for ∼70% determined by integration of the scattering intensity from 0–35° (Figure S25). The analogous GIWAXS pattern collected for *S*-P3­(1EH)­EF is isotropic (Figure 5). Neither drop-casting nor solvent annealing from CHCl_3_ or THF affords anisotropic scattering patterns (Figure S24). The decreased intensity for the signals attributed to side-chain packing in the mid-*q* range in the powder XRD (Figure 5), the increase in π-stacking distance from the achiral to chiral derivative (from 3.39 to 3.50 Å), and the weak isotropic scattering pattern for *S*-P3­(1EH)­EF in GIWAXS are clear indications of the decreased order.

^36^ Given the increased disorder for *S*-P3­(1EH)­EF in the solid state, we anticipated this could lead to a reduction in melting temperature, similar to what has been observed with poly­(3-hexylthiophene) and poly­(3-(2-ethylhexyl)­thiophene).^46^ XRD analysis at different temperatures was carried out where the polymer samples were packed in a metallic washer, with both ends sealed using Kapton tape (Figure S16).

For P3HEF, the polymer maintained its π-stacking (∼1.86 Å^–1^) and low *q* (∼0.3 Å^–1^) peaks even as the temperature was increased to 280 °C (Figure 6 – Left). These peaks shift slightly toward higher *q* upon heating but eventually return to their original positions with sharper peaks (Figure 6 - Left), suggesting some degree of annealing at higher temperatures. No signs of melting were noted for P3HEF up to 350 °C (Figure S21), nor any discernible change in the physical appearance of the polymer. In contrast, upon heating *S*-P3­(1EH)­EF, the peak centered around 1.8 Å^–1^, identified as the π-stacking distance, decreased in intensity and shifted toward lower *q*, consistent with an expansion of the π-stacking distance (Figure 6 – Right). Simultaneously, there was a decrease in intensity observed in the low *q* peak and the mid-*q* region. Near 155 °C, the π-stacking peak completely disappeared, accompanied by a reduction in intensity for the sharp low *q* peak around 0.3 Å^–1^. Upon cooling, the π-stacking peak reappeared, accompanied by a concurrent increase in the intensity of the low *q* peak (albeit weaker than previous). Importantly, the peaks appeared broader and exhibited a slight shift relative to the original positions. Comparing the intensity of the scattering pattern before and after melting is difficult because when the polymer melts, it becomes fluid, leading to variability in the mass balance of polymer within the X-ray beam’s position, which impacts signal intensity (Figure S20).

**Figure 6:**
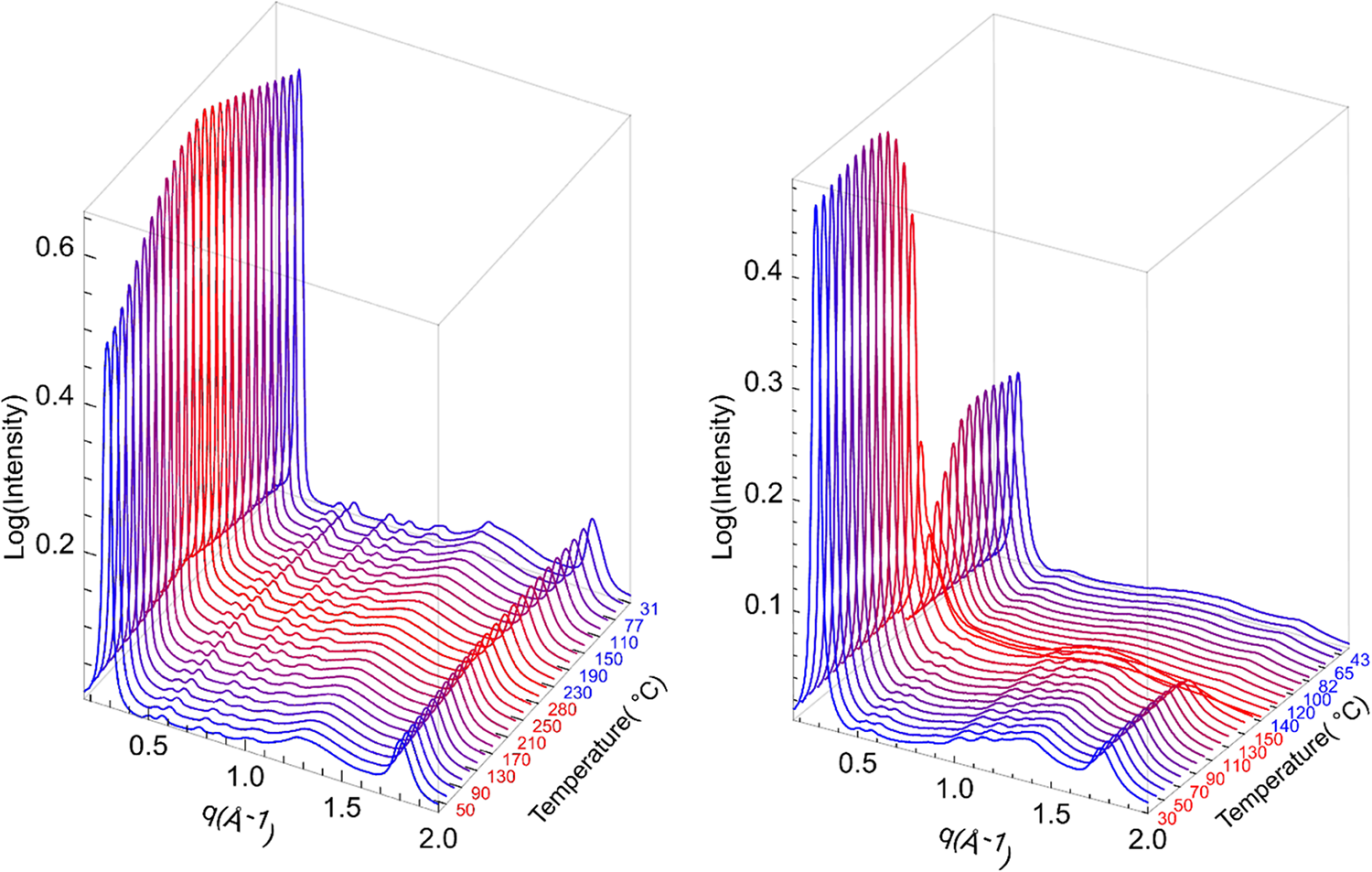
Powder X-ray scattering patterns for P3HEF (*M*_*n*_ = 8.2 kg/mol, *Đ* = 1.28, left) and *S*-P3­(1EH)­EF (*M*_*n*_ = 14.9 kg/mol, *Đ* = 1.36, right) collected at different temperatures.

Differential scanning calorimetry (DSC) data shown in Figures S28–S32 are indicative of the same behavior. No melt transition was observed for P3HEF upon heating from −50 to 220 °C (Figures S28–S29), while a melt transition was observed at 141 °C for *S*-P3­(1EH)­EF (Figures S30–S32). Notably, during heating the second run, an exothermic crystallization transition (cold crystallization) was observed at 115 °C for *S*-P3­(1EH)­EF, which merged with the subsequent melting transition. In the cooling cycle, no distinct crystallization transition was observed, with only a broad and low-intensity exothermic event detected (Figure S31).

The nanoscale morphology of P3HEF was examined using atomic force microscopy previously and filamentous aggregates were observed.^36^ Additional insights into morphology were obtained for P3HEF and *S*-P3­(1EH)­EF using optical microscopy and scanning electron microscopy (SEM) of deposits prepared from THF and CHCl_3_ solutions drop-cast onto silicon wafers (Figures S42–S45). Clear formation of aggregates was noted at all concentrations, with the size being dependent on the concentration of the polymer solution (larger aggregates from more concentrated solutions). Drop-casting of P3HEF from THF or CHCl_3_ did not markedly influence the size and shape of the structures but for *S*-P3­(1EH)­EF, films were more homogeneous from CHCl_3_, and worm-like aggregates were visible when drop-cast from 1 and 0.5 mg/mL solutions (Figure S45).

### Optical Spectroscopy

Motivated by the melting behavior observed for *S*-P3­(1EH)­EF in the solid state (Figure 5), we investigated whether the chiral polymer undergoes temperature-dependent conformational changes in solution. Three solvents (CHCl_3_, THF, and *n*-octane) were chosen to establish a solvent progression from good to poor solvents for the *S*-P3­(1EH)­EF polymer. This allowed us to probe how solvent influences the stability of the folded helical conformation at room temperature and under heating.

In CHCl_3_ at room temperature, *S*-P3­(1EH)­EF is partially unfolded, as indicated by two absorption bands at 335 and 445 nm and minimal circular dichroism (CD) response (Figure S13).^36,37^ Variable-temperature absorption and CD spectra collected in CHCl_3_ over the range of 10–50 °C were nearly identical, suggesting no temperature-induced conformational changes for the polymer (Figures S10 and S12). In *n*-octane, the polymer adopts a helical conformation that remains stable across the same temperature range, as evidenced by the strong CD signal and lack of π–π* absorption in the absorption spectrum (Figures S9 and S11).^37^

Upon dissolution of *S*-P3­(1EH)­EF in THF at room temperature, the absorption spectrum closely resembled that of achiral P3HEF (Figure 7A1) suggesting a helical conformation. A change was noted in THF, as evidenced by the near complete loss of CD signal just above room temperature (purple and red spectra in Figure 7A2,7A3). This coincides with the appearance of the π–π* band near 450 nm in the absorption spectrum (Figure 7A1). This behavior is reversible, and the CD signature reappears upon cooling, suggesting the folded helical conformation can be “melted” just above 30 °C when dissolved in THF. Variable-temperature NMR studies of *S*-P3­(1EH)­EF in THF-*d*_8_ provided further evidence for this melting behavior, with sharpening of signals above 30 °C and broadening upon cooling to room temperature and below (Figure S7). The differential absorption of left- and right-handed circularly polarized light was quantified by determining the dissymmetry factor (*g* = Δε/ε where ε is the extinction coefficient). This value is concentration independent, and a *g*_abs_ maximum of ∼0.04 was noted around 515 nm in THF and *n*-octane (Figures 7A3 and S11).

**Figure 7:**
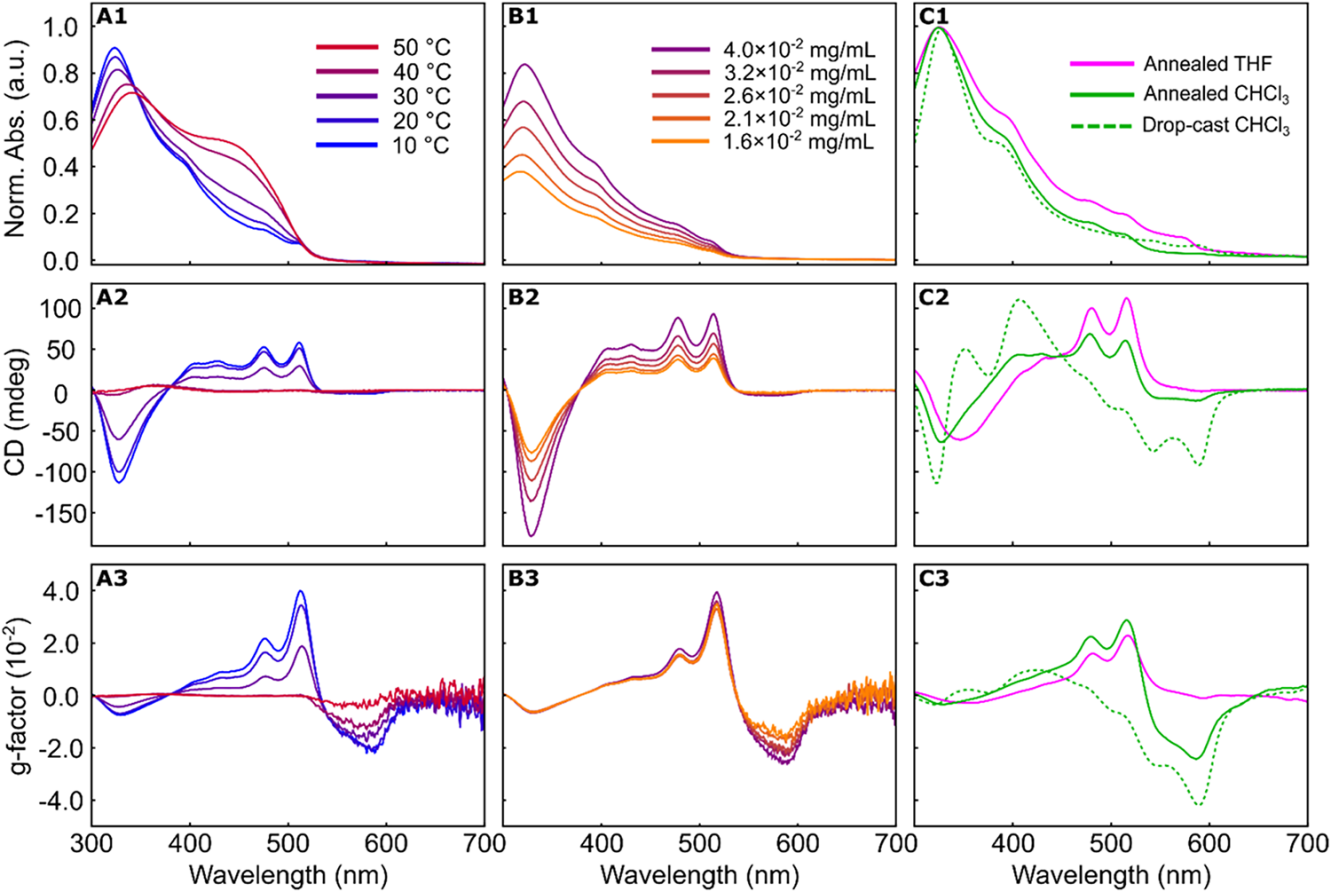
UV–vis absorption (top), circular dichroism (middle), and *g*-factor (bottom) spectra of *S*-P3­(1EH)­EF. (A1, A2, A3) Temperature-dependent spectra for *S*-P3­(1EH)­EF (*M*_*n*_ = 14.9 kg/mol, *Đ* = 1.36) in THF from 10–50 °C at ∼0.03 mg/mL. (B1, B2, B3) Spectra collected at different concentrations for *S*-P3­(1EH)­EF (*M*_*n*_ = 19.4 kg/mol, *Đ* = 1.19) in THF (0.04–0.016 mg/mL). (C1, C2, C3) Solid-state absorption spectra for *S*-P3­(1EH)­EF (*M*_*n*_ = 19.4 kg/mol, *Đ* = 1.19) from THF (magenta) and CHCl_3_, both drop-cast (dotted green) and annealed (solid green).

To support the claim that the chiral *S*-P3­(1EH)­EF adopts a single-chain helix in THF and that supramolecular helical aggregates are not forming, concentration studies and dynamic light scattering (DLS) measurements were carried out (Figures 7B and S14). If the polymer adopts a single-chain helix, the CD and absorbance intensity would be expected to decrease upon dilution, but the spectral pattern should remain the same. This is consistent with our observations on concentrations ranging from 0.04–0.016 mg/mL (Figure 7B). Additionally, at much higher concentrations (1 mg/mL in THF), the volume-averaged hydrodynamic diameter (*D*_H_) distribution recorded for *S*-P3­(1EH)­EF is 8.4 ± 0.3 nm (Figure S14). The *D*_H_ for the same polymer sample at 1 mg/mL in CHCl_3_ is within 1 nm (7.3 ± 0.4 nm, Figure S15). The small particle size and narrow distributions suggest well-defined particles in both solvents.

### Solid-State Absorption and CD

Drop-cast thin films of *S*-P3­(1EH)­EF (*M_n_* = 19.4 kg/mol) were prepared from CHCl_3_ and THF for solid-state CD studies (Figure 7C). The absorption spectra obtained when cast from both polymers are quite similar, with subtle differences in fine structure observed on the low energy tail (Figure 7C1). The long tail again provides evidence of the helical conformation in the solid state. When comparing the CD spectra, significantly higher intensity is noted between 525 and 600 nm for the samples prepared from CHCl_3_. This is particularly notable in the dissymmetry factor spectra from CHCl_3_ (annealed and drop-cast are shown as solid and dotted green traces in Figure 7C3). This can be partially explained by the polymer behavior in each of those solvents, as preaggregation of *S*-P3­(1EH)­EF in CHCl_3_ where it is partially unfolded will be different compared to THF, where the polymer retains the helical conformation. It points to the sensitivity of this polymer to the solvent environment. We also noted that *S*-P3­(1EH)­EF drop-cast from THF has a lower maximum *g*_abs_ value relative to that noted in solution between 510 - 515 nm (*g*_abs_ solution ∼0.04 versus *g*_abs_ solid ∼0.02). Measurements at different incident angles and from both the front and back sides of the film were carried out to account for effects of linear dichroism and linear birefringence, and no significant differences were observed in either the shape or intensity of the spectra when changing the angle.^2^

## Conclusions

The results presented here demonstrate that computational analysis can be a valuable tool for predicting the helix handedness of P3AEFs, a task that is challenging with many synthetic helical polymers.^47^ In the solid state, the mutability of the chiral helical polymer due to the branched 1-ethylhexyl side chain was clear from the observed melting behavior in X-ray diffraction and DSC studies. While the achiral P3HEF does not melt up to 350 °C, *S*-P3­(1EH)­EF melts near 150 °C (141 °C by DSC), consistent with increased disorder and more frustrated packing caused by the branched chiral side chain. A key objective of future work will be to examine how packing and organization of these helical structures are influenced by different chiral side chains, as well as to compare these materials with cyclo­[*n*]­furans^48^ bearing chiral side groups.

## Supplementary Material





## Data Availability

The data underlying this study are available in the published article and its Supporting Information

## Notes

Experimental procedures, experimental setup, NMR data, gel permeation chromatography (GPC) data, differential scanning calorimetry (DSC) traces, dynamic light scattering (DLS) data, X-ray scattering patterns (PDF)
Optimized geometries of computed structures in xyz format (ZIP)
