# Supplementary material for: Examining the Impact of Side-Chain Chirality on Conformation of a Helical Poly(3‑(S‑1-ethylhexyl)esterfuran)
Source: Macromolecules. 2025 Nov 15;58(22):12336–45. doi: 10.1021/acs.macromol.5c01499 (PMC13011260; doi:10.1021/acs.macromol.5c01499)
Supplement: Supplementary file 1 [file ma5c01499_si_001.pdf]

Supporting Information

Examining the Impact of Side-Chain Chirality on Conformation of a Helical Poly(3-(*S*-1-ethylhexyl)esterfuran)

Dhruv Sharma, Manami Kawakami, Megan Rice, Erin Smith, Soren Westrey, Yuyang Shang, Claire Gist, [¶] *Leticia Maria Pequeno Madureira, Karl H. G. Schulz, Anthony J. Varni, Isabella M. Stranick, Roberto R. Gil, Stephanie Tristram-Nagle,*[¶] *Linda Peteanu,* Tomasz Kowalewski,* Kevin J. T. Noonan**

Department of Chemistry, Carnegie Mellon University, 4400 Fifth Avenue, Pittsburgh, Pennsylvania 15213, United States

[¶]Physics Department, Carnegie Mellon University, Pittsburgh, Pennsylvania 15213-2617, USA

Table of Contents

Materials and Methods..S4-S6

Experimental Procedures for *S*-P3(1EH)EF and P3HEF Synthesis S7-S8

- **Scheme S1.** General Synthetic Route for Polyfuran S7
- **Table S1.** Polymerization Conditions and Yields for *S*-P3(1EH)EF S7

GPC Traces of *S*-P3(1EH)EF and P3HEF **(Figure S1)**.. S9

NMR Spectra of *S*-P3(1EH)EF and P3HEF **(Figure S2-S6)**...S10-S12

Variable Temperature NMR Spectra of *S*-P3(1EH)EF and P3HEF **(Figure S7-S8)** S13

Variable Temperature Absorption/CD Spectra of *S*-P3(1EH)EF **(Figure S9-S12)** S14-S15

Absorption/CD Spectra of *S*-P3(1EH)EF in CHCl3, *n*-octane and THF **(Figure S13)**............. S16

Dynamic Light Scattering (DLS) Measurements **(Figures S14–S15)** S17

Variable Temperature (VT) Powder Diffraction Setup **(Figure S16)** S18

Powder Diffraction Patterns of *S*-P3(1EH)EF and P3HEF **(Figure S17-S19)**....................S19-S20

Variable Temperature Powder Diffraction Studies **(Figure S20–S21)** S21

2D Images of Diffraction Patterns of *S*-P3(1EH)EF and P3HEF **(Figure S22-S23)** S22

GIWAXS 2D Images of *S*-P3(1EH)EF on Glass Slides **(Figure S24)**...................................... S23

Azimuthal Intensity Profiles of P3HEF and *S*-P3(1EH)EF **(Figures S25)** S24

Thermogravimetric Analysis (TGA) **(Figures S26–S27)**.. S25

Differential Scanning Calorimetry (DSC) Analysis **(Figure S28–S32)** S26-S28

Predicted Absorption and CD Spectra for P3MEF (13-mers) **(Figure S33)**.............................. S29

Natural Transition Orbitals (NTO) for P3MEF (13-mers) **(Figures S34–S39)** S29-S32

Predicted absorption for varying proportions of *syn* and *anti* linkages **(Figure S40)** S32

Helix-sense bias vs. oligomer length (6-13-mers) **(Figure S41)** S33

Optical microscopy and SEM images of P3HEF and *S*-P3(1EH)EF **(Figures S42–S45)**..... S34-36

References S37

Materials and Methods. All reactions and manipulations of air and water-sensitive compounds were carried out under a dry N_2 atmosphere using a mBraun glovebox or standard Schlenk techniques with dried and degassed solvents. All solvents and chemicals used for extraction and column chromatography were used as received. Flash chromatography was completed using a Biotage Isolera One Flash Chromatography System with 250-400 mesh silica gel (grade 60). All reagents were obtained from commercial sources and used as received. (*S*)-octan-3-yl 2-bromo-5-(4,4,5,5-tetramethyl-1,3,2-dioxaborolan-2-yl)furan-3-carboxylate was prepared according to a literature procedure.[1]

NMR Analysis. All NMR experiments were collected at 300 K on either a two-channel Bruker Avance III NMR instrument equipped with a Broad Band Inverse (BBI) probe, or a Bruker NEO 500 NMR spectrometer equipped with the multinuclear BBO Prodigy cryoprobe. Both instruments operate at 500 MHz for ^1H. The ^1H NMR spectra are referenced to residual protio solvents (7.26 ppm for $CDCl_3$).

Gel-Permeation Chromatography. GPC measurements were performed on a Waters Instrument equipped with an e2695 autosampler, a Waters 2414 refractive index (RI) detector, and two SDV columns (Porosity 1000 and 100000 Å; Polymer Standard Services) with THF as the eluent (flow rate of 1 mL/min, 40 °C). A 9-point calibration based on polystyrene standards (Polystyrene, ReadyCal Kit, Polymer Standard Services) was applied for the determination of molecular weights.

UV-Vis Spectroscopy. UV-vis spectra of all polymers were recorded on an Agilent 8453 spectrophotometer with an attached temperature controller. Prior to recording the spectra for all polymers, a 100% transmittance sample was taken of the cuvette (quartz, 10 mm × 10 mm). The "blank" of the solvent ($CHCl_3$, THF, *n*-octane) was then collected for baseline subtraction during analysis. Solution measurements were completed using $CHCl_3$, THF, or *n*-octane as the solvents.

Circular Dichroism (CD) Spectroscopy. For solution measurements, spectra of all polymers were

recorded on a Jasco J-715 spectropolarimeter. Prior to recording the spectra for all polymers, a 100% transmittance sample was taken of the cuvette (quartz, 10 mm × 10 mm). The "blank" of the solvent was then collected for baseline subtraction during analysis.

For solid measurements, the polymer solution was cast from THF or $CHCl_3$ onto separate microscope glass slides (10 mm × 30 mm) using two different methods: drop casting and solvent annealing. Each method was applied independently to create distinct thin films. The absorption and CD spectra of these films were then recorded using a JASCO 1500 CD spectrometer at 25 °C. A spectra of "blank" glass slide was also collected for baseline subtraction during analysis. The detailed casting procedure is provided in the next section, X-ray Diffraction Studies.

X-ray Diffraction Studies. Powder and Grazing Incidence Wide Angle X-ray Scattering (GIWAXS) measurements were conducted using a Xeuss 3.0 (XENOCS, Holyoke, MA) instrument, equipped with a CuKα rotating anode source (λ ~ 1.5418 Å) and an Eiger 1M detector (Dectris, Switzerland). All measurements were performed with the instrument operated at 40 kV and 30 mA. The configuration was set to *Standard* with absolute intensity enabled. The sample-to-detector distance was fixed at 100 mm for all experiments. Exposure times were 600 seconds for powder studies and 120 seconds for variable temperature measurements. Scattering intensity (I) *versus* scattering vector q ($q = 4\pi/\lambda \sin(\theta)$, where λ is the wavelength and 2θ is the scattering angle) was obtained by azimuthally averaging the 2D data. A Linkam HFSX350 stage was utilized to achieve controlled heating of the sample.

For powder diffraction studies, polymer samples were prepared by slow evaporation from a 2 mg/mL solution in either $CHCl_3$ or THF at room temperature. The samples were then dried in a vacuum oven at 50 °C for one hour.

Both SiO_2 wafers (10 × 5 mm) and microscope glass slides (10 mm × 30 mm) served as substrates for casting thin films for GIWAXS, absorbance, and CD studies. Prior to casting, the substrates were cleaned with hexanes, acetone, and isopropanol, followed by drying with N_2. A 2 mg/mL polymer solution in HPLC grade solvent was prepared, heated to 50 °C for 15 minutes in a 20 mL scintillation vial equipped with a Teflon screw cap, filtered through a 0.22 μm PTFE syringe filter, and reheated at 50 °C for 5 minutes before use.

For solvent annealing, the cleaned substrates were placed in a petri dish lined with 1 mL of the casting solvent. The heated polymer solution was poured onto the substrates to cover them completely, and the dish was covered with a lid to allow slow drying by solvent-vapor annealing. For drop casting, the cleaned substrates were placed in a petri dish. The heated polymer solution was poured onto the substrates to cover them completely and then left to dry without a lid. All procedures involving volatile organic solvents were carried out in a well-ventilated laboratory environment following standard chemical safety protocols.

Dynamic Light Scattering (DLS). Malvern Zetasizer Ultra-Red instrument was used to determine the hydrodynamic diameter of *S*-P3(1EH)EF polymer particles in solution. Anhydrous $CHCl_3$ and THF (spectrophotometric grade, ≥99.8%) were used from freshly opened bottles.

Computation. Calculations for the regioregular α-linked head-to-tail methyl furan-3-carboxylate 13-mers and 6-mers were carried out first by optimization of the structure using the B3LYP-D3(BJ) functional and a 6-31G(d,p) basis set, followed by time-dependent DFT (TD-DFT) calculations using the CAM-B3LYP/6-31G(d,p) functional to predict absorption and CD spectra. All calculations/optimizations were completed using a Polarizable Continuum Model (PCM) with the integral equation formalism variant (IEFPCM) and CH_2Cl_2 as the solvent. Molecular optimizations of structures with the 1-ethylhexyl side chain ranging from 6 to 14 units were performed with GFN2-xTB. Optimized geometries for all relevant structures are included as a compressed folder with relevant .xyz files.

Scheme S1. General synthetic route for polyfuran using a Suzuki–Miyaura type monomer.

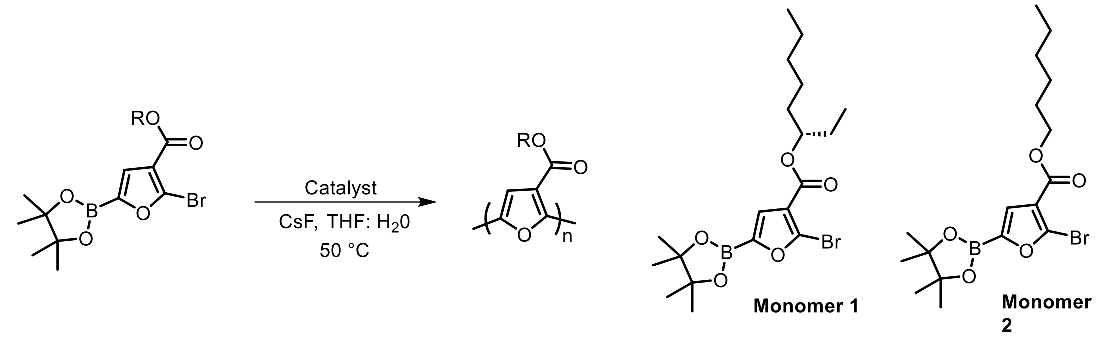

***S*-P3(1EH)EF** could be synthesized using the Pd-PEPPSI-IPent catalyst.[2] In a N_2 filled glovebox, a 20 mL scintillation vial equipped with a Teflon screw cap was charged with PEPPSI-IPent, CsF, Monomer 1, and THF (amounts listed in Table S1). The reaction vial was capped, removed from the glove box, and placed in a 60 °C oil bath. A small amount of degassed H_2O (listed in Table S1) was injected into the reaction with a N_2 purged syringe. After 3 h, the polymerization was quenched using a 6 M methanolic HCl solution. The precipitate was collected using vacuum filtration and then washed with methanol, water, and hot acetone to remove any unreacted monomers, oligomers, and salts, respectively. The resultant solid was dissolved in a minimal amount of THF and reprecipitated with methanol. The precipitate was collected using vacuum filtration and dried *in vacuo*. The final polymer was obtained as a dark-red solid for *S*-P3(1EH)EF.

Table S1. Polymerization Conditions and Yields for *S*-P3(1EH)EF.

***S*-P3(1EH)EF**	**Monomer 1**	**PEPPSI-IPent**	**CsF**	**THF**	**H_2O**	**Yield (%)**
**M_n = 14.9 kg/mol** **$Đ$ = 1.36**	400 mg (0.93 mmol)	36.9 mg (4.7×10^{-2} mmol)	424.7 mg (2.80 mmol)	25.9 mL	5.2 mL	156 mg (75 %)
**M_n = 19.4 kg/mol** **$Đ$ = 1.19**	200 mg (0.47 mmol)	18.4 mg (2.3×10^{-2} mmol)	212.4 mg (1.40 mmol)	12.9 mL	2.6 mL	82 mg (78 %)

P3HEF was synthesized similarly to a previous report.[1] In a N_2 filled glovebox, a 20 mL scintillation vial equipped with a Teflon screw cap was charged with $NiCl_2$(IPr)(PPh_3) (4.86 mg, 0.6×10^{-2} mmol), CsF (283.9 mg, 1.87 mmol), Monomer 2 (250 mg, 0.62 mmol), and THF (20.0 mL). The reaction vial was capped, removed from the glovebox, and placed in a 50 °C oil bath. After 5 minutes of stirring, a small amount of degassed H_2O (191 μL) was injected into the reaction with a N_2 purged syringe. After 1 hour, the polymerization was quenched using 6 M methanolic HCl solution. The precipitate was collected using vacuum filtration, then the crude solid was washed with methanol, water, and hot acetone to remove any unreacted monomer, oligomers, and salts, respectively. The resultant solid was dissolved in a minimal amount of THF, and reprecipitated with methanol. The precipitate was collected using vacuum filtration and dried *in vacuo*. The final polymer was obtained as a dark-red solid, 112 mg (93% yield), M_n = 8.2 kg/mol, $Đ$ = 1.28. ^{1}H NMR data matched with the previous report.[1]

GPC traces of *S*-P3(1EH)EF and P3HEF

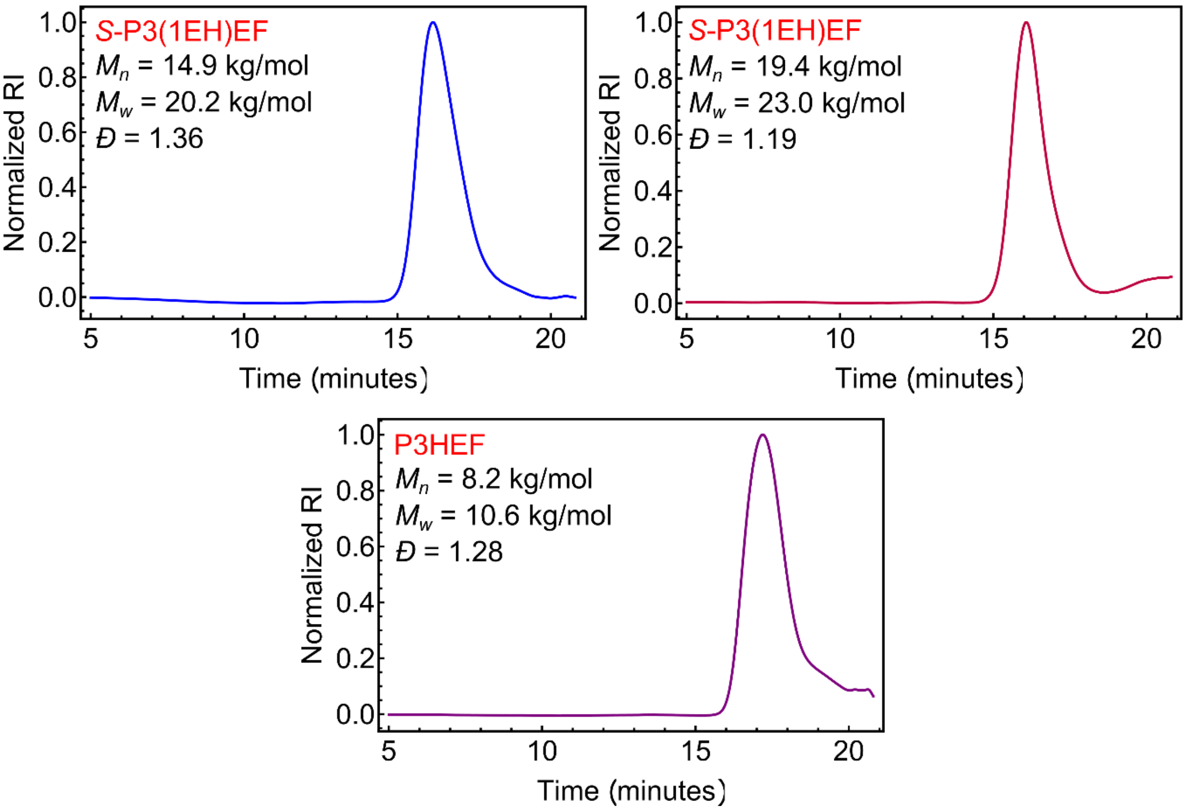

Figure S1. GPC traces of *S*-P3(1EH)EF and P3HEF.

^{1}H-NMR spectra of *S*-P3(1EH)EF and P3HEF

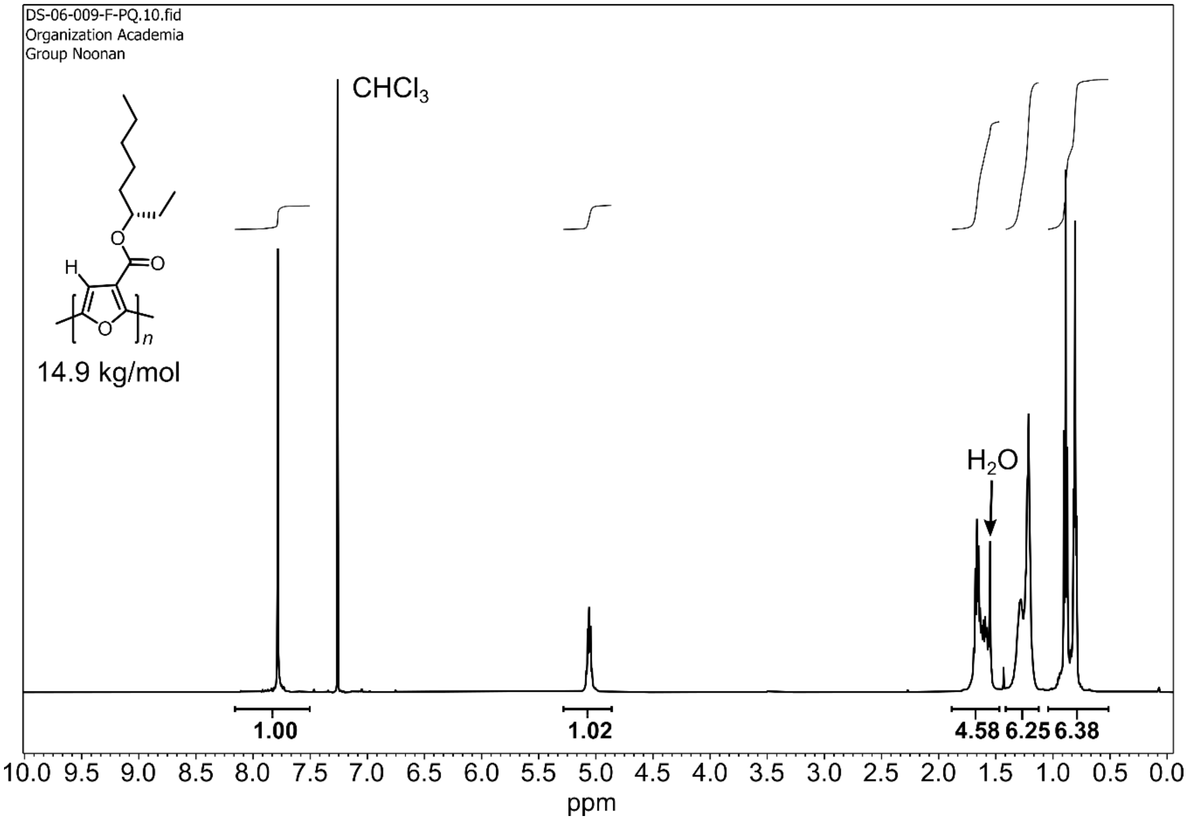

Figure S2. ^{1}H-NMR spectrum of *S*-P3(1EH)EF (M_n = 14.9 kg/mol) collected in $CDCl_3$.

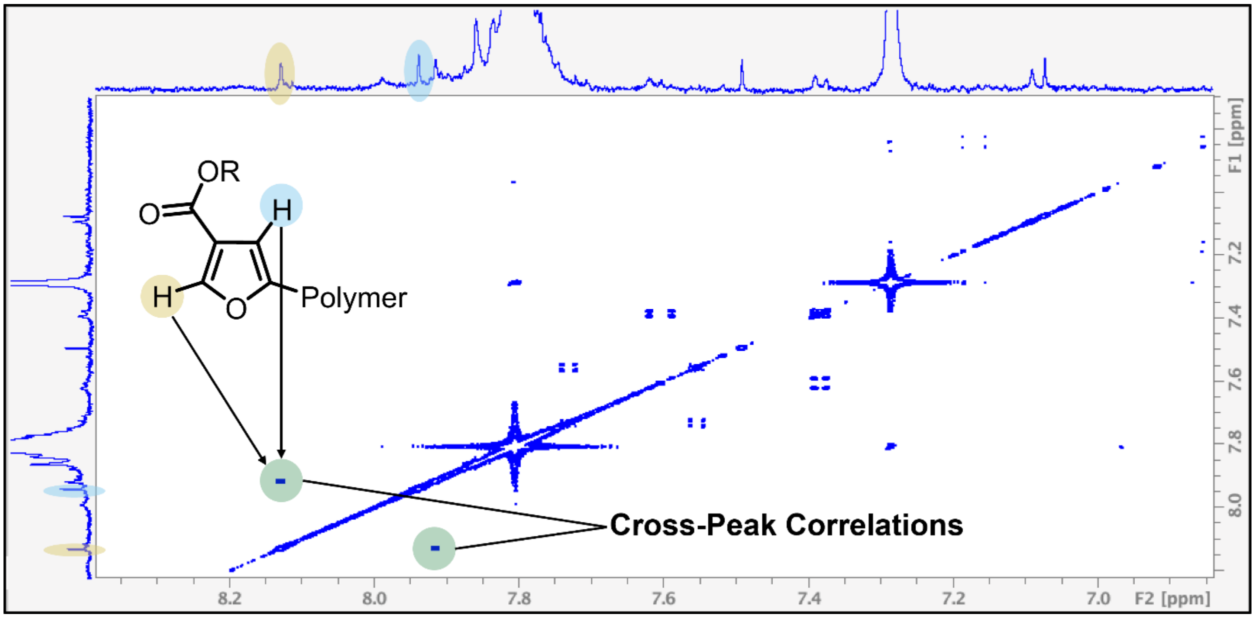

Figure S3. ^{1}H-^{1}H 2D COSY spectrum of *S*-P3(1EH)EF (M_n = 14.9 kg/mol) collected in $CDCl_3$.

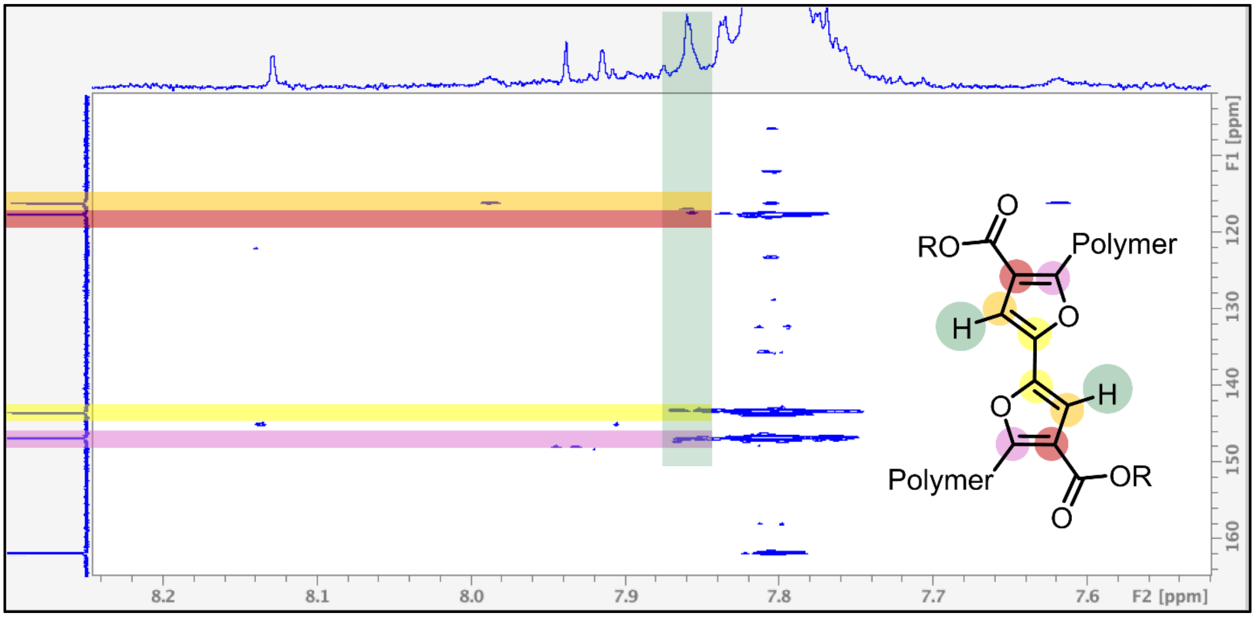

Figure S4. ^{1}H-^{13}C 2D HMBC spectrum of *S*-P3(1EH)EF (M_n = 14.9 kg/mol) collected in $CDCl_3$.

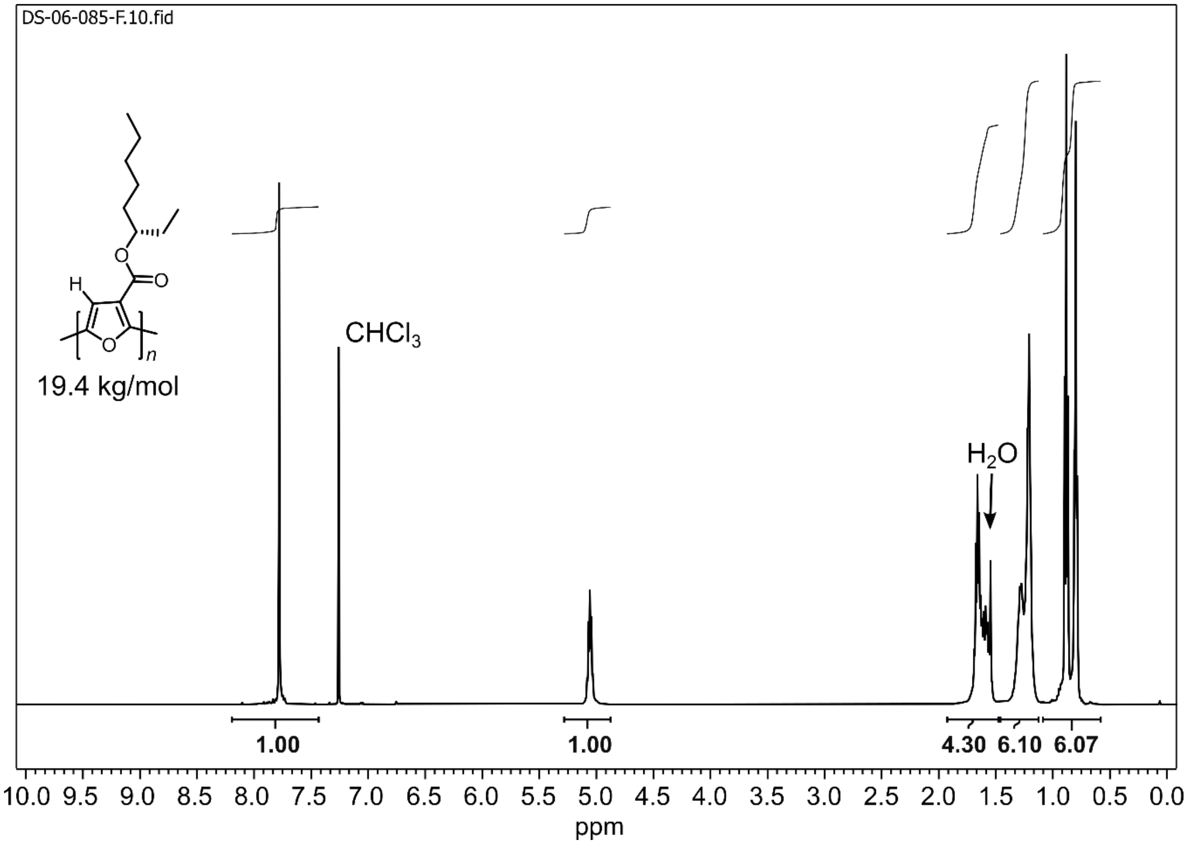

Figure S5. ^{1}H-NMR spectrum of *S*-P3(1EH)EF (M_n = 19.4 kg/mol) collected in $CDCl_3$.

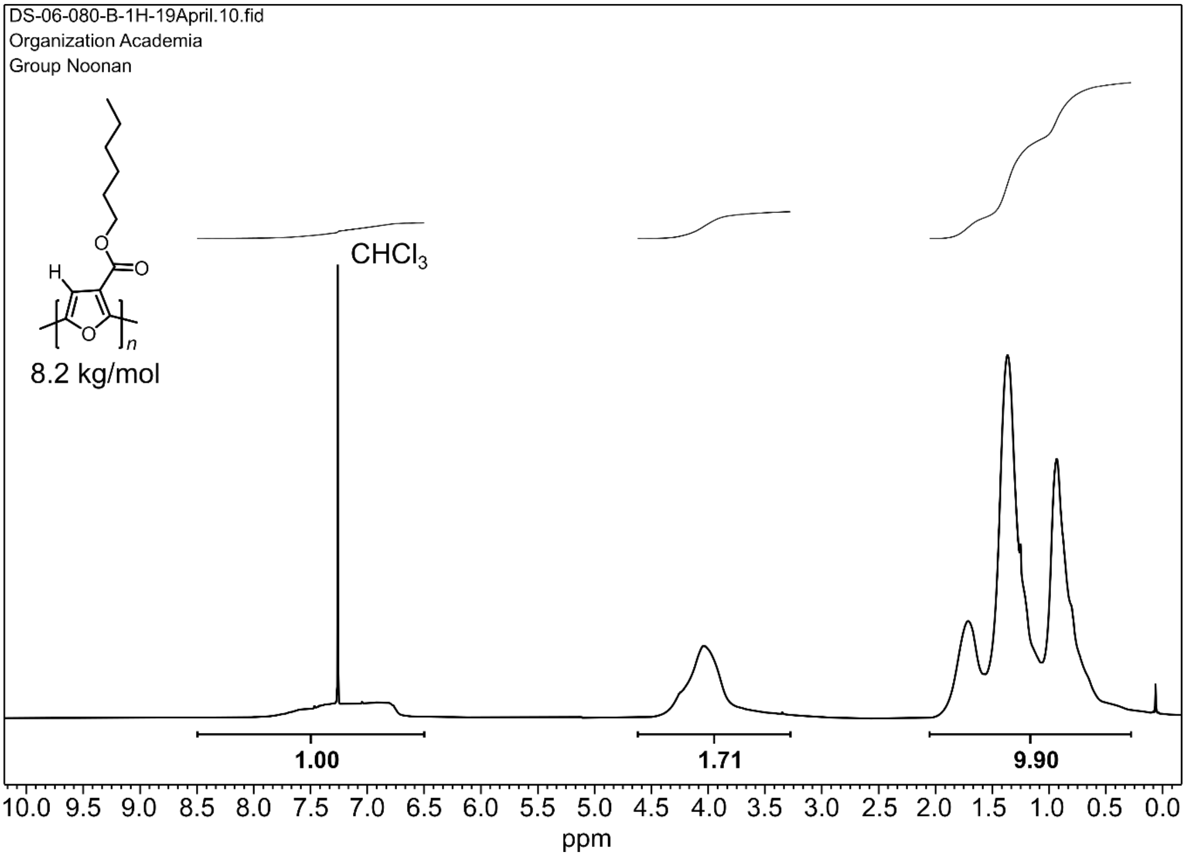

Figure S6. ^{1}H-NMR spectrum of P3HEF (M_n = 8.2 kg/mol) collected in $CDCl_3$.

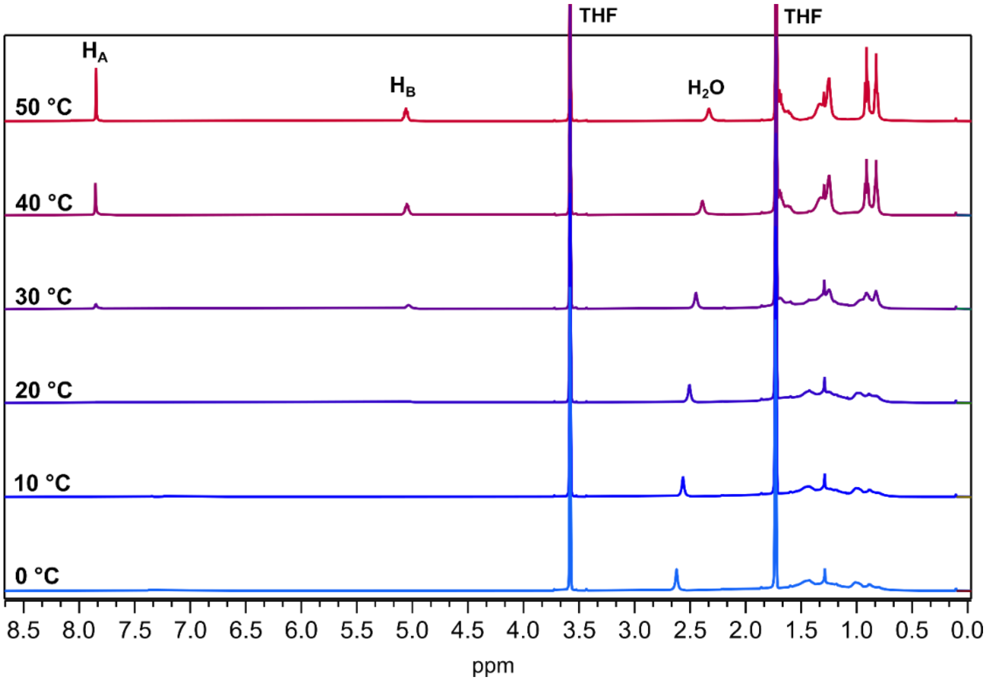

Figure S7. Temperature-dependent ^{1}H-NMR spectra of *S*-P3(1EH)EF (M_n = 19.4 kg/mol) collected in THF-d_8.

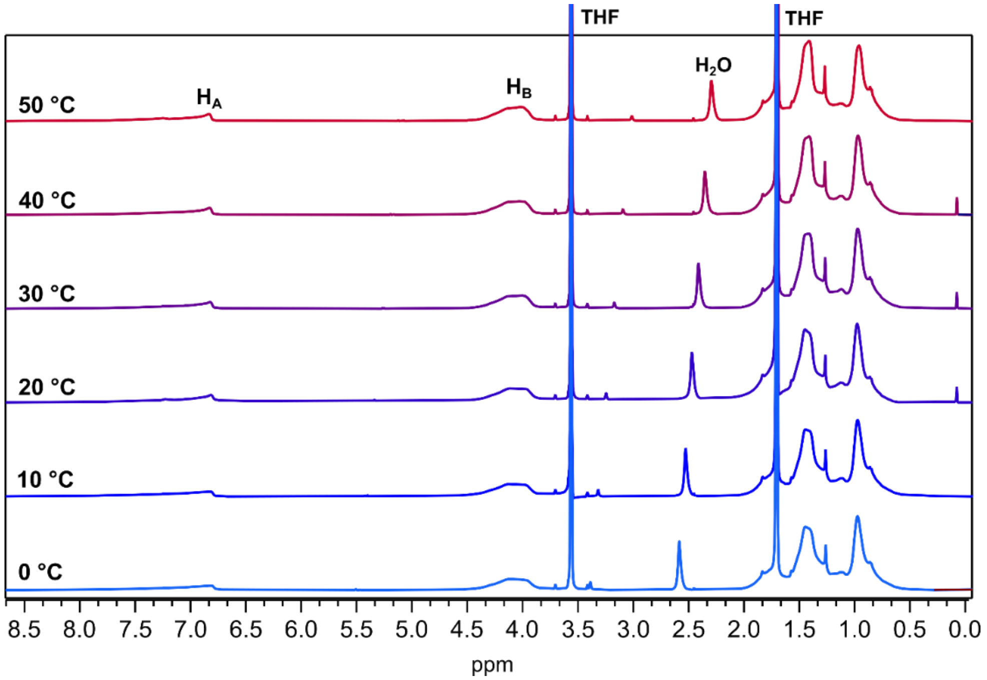

Figure S8. Temperature-dependent ^{1}H-NMR spectra of P3HEF (M_n = 8.2 kg/mol) collected in THF-d_8.

Variable Temperature UV-Vis

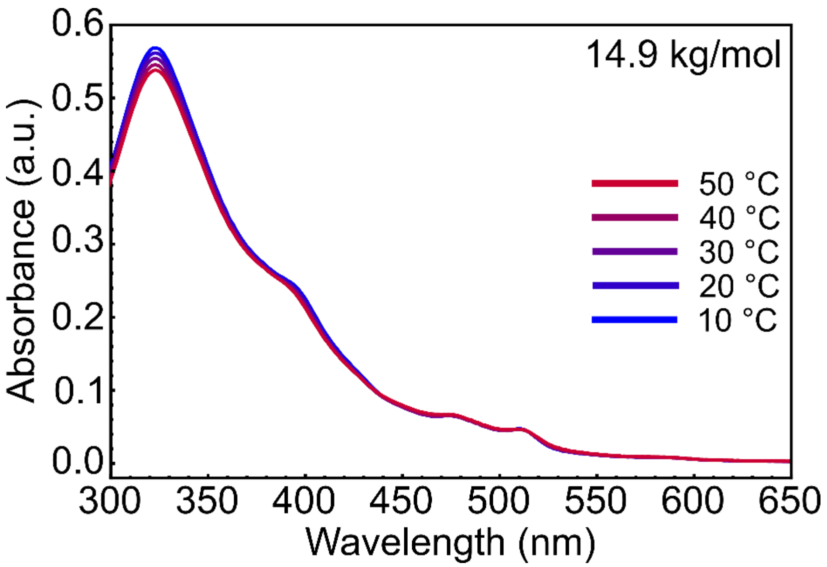

Figure S9. Variable temperature UV-Vis spectra of *S*-P3(1EH)EF (M_n = 14.9 kg/mol) collected in *n*-octane over 10-50 °C.

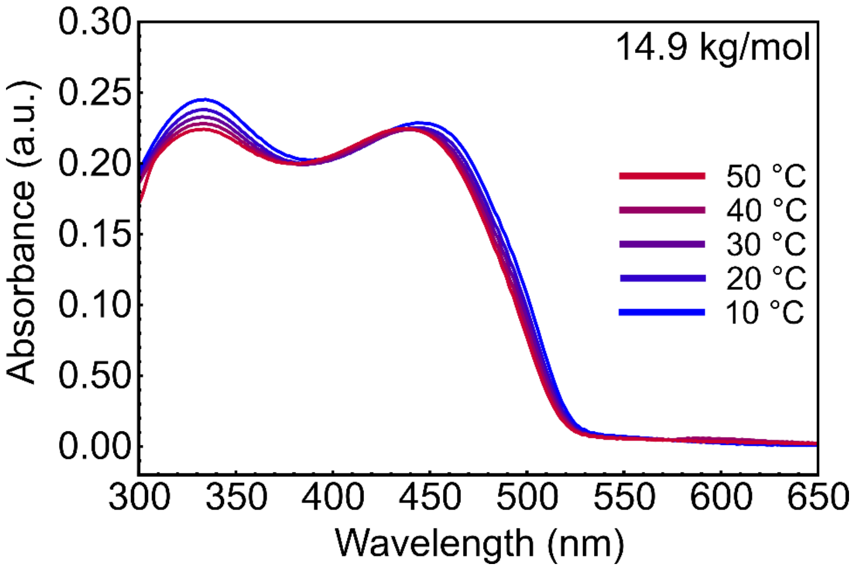

Figure S10. Variable temperature UV-Vis spectra of *S*-P3(1EH)EF (M_n = 14.9 kg/mol) collected in $CHCl_3$ over 10-50 °C.

Variable Temperature CD spectra

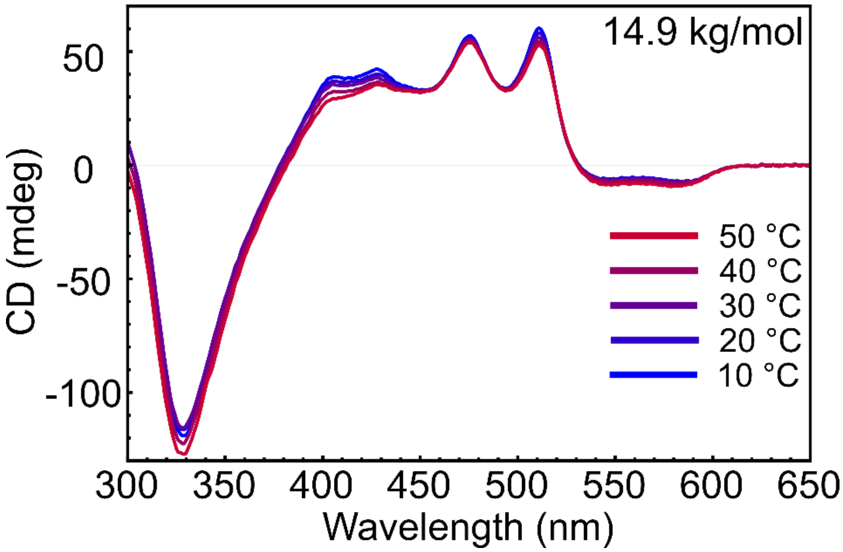

Figure S11. Variable temperature CD spectra of *S*-P3(1EH)EF (M_n = 14.9 kg/mol) collected in *n*-octane over 10-50 °C.

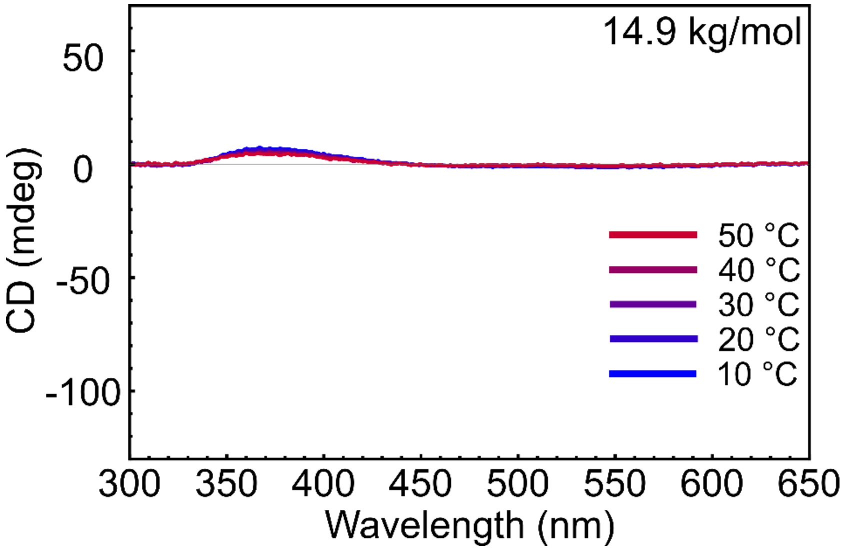

Figure S12. Variable temperature CD spectra of *S*-P3(1EH)EF (M_n = 14.9 kg/mol) collected in $CHCl_3$ over 10-50 °C.

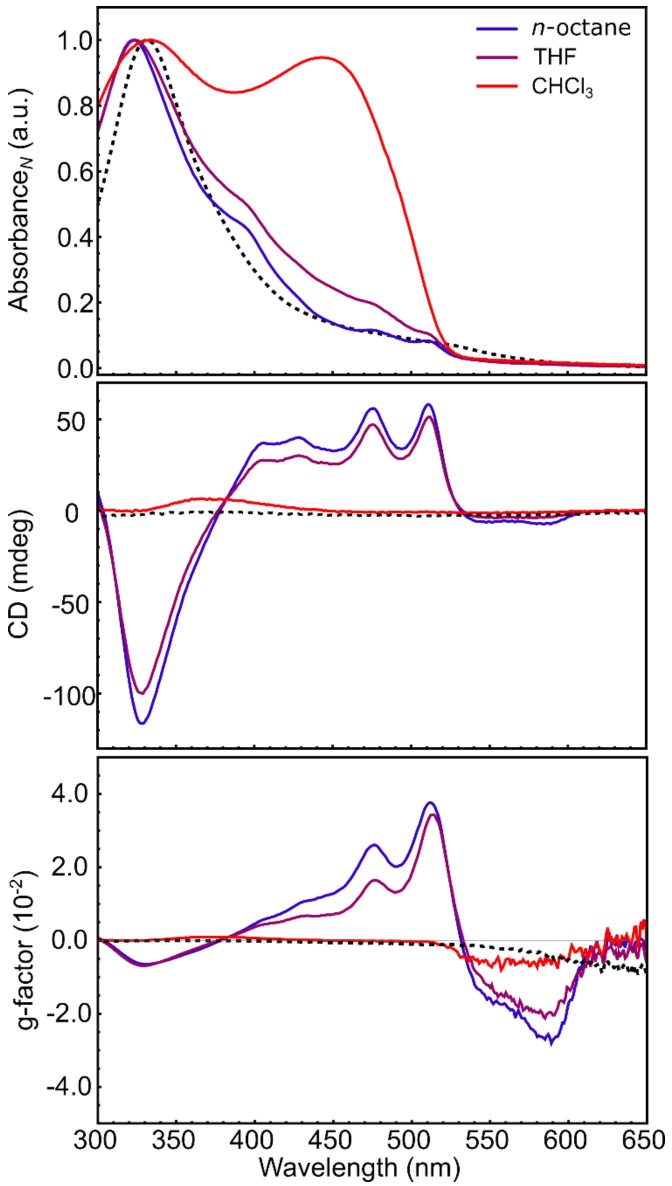

Figure S13. UV−Vis absorption (top), circular dichroism (middle), and g-factor (bottom) spectra of *S*-P3(1EH)EF (M_n = 14.9 kg/mol, $Đ$ = 1.36) in $CHCl_3$ (red), *n*-octane (blue), and THF (purple). The dotted black line corresponds to P3HEF (M_n = 8.2 kg/mol, $Đ$ = 1.28). All solutions were prepared at an approximate concentration of 0.015 mg/mL, and the measurements were conducted at ambient temperature (~22 °C).

Dynamic Light Scattering (DLS)

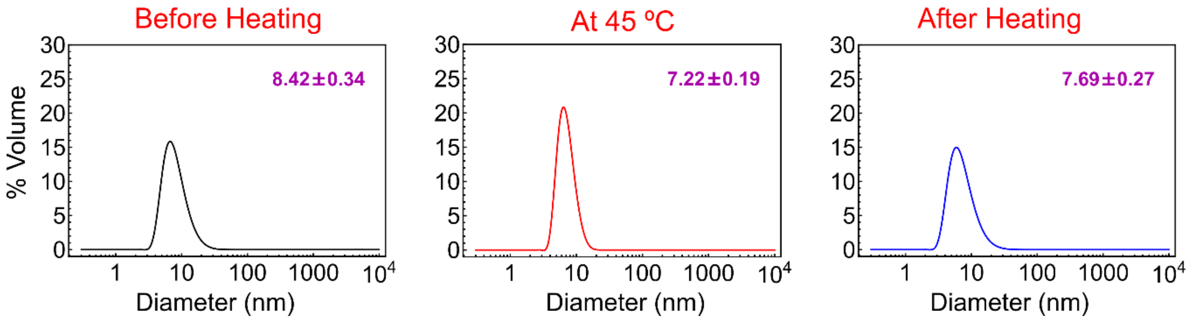

Figure S14. Dynamic Light Scattering (DLS) measurement of *S*-P3(1EH)EF (M_n = 14.9 kg/mol) solution (1 mg/mL) in THF. The volume-averaged hydrodynamic diameter (<D_H>) is shown in magenta. The solution was heated to 45 °C to assess any significant reduction in particle size.

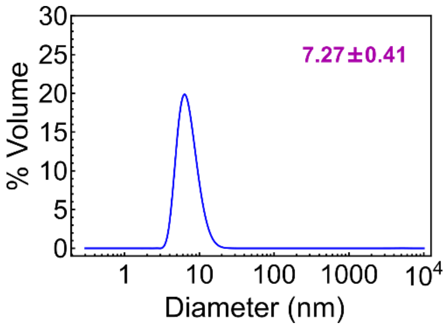

Figure S15. Dynamic Light Scattering (DLS) measurement of *S*-P3(1EH)EF (M_n = 14.9 kg/mol) solution (1 mg/mL) in $CHCl_3$. The volume-averaged hydrodynamic diameter (<D_H>) is shown in magenta. The measurement was collected at 25 °C.

Setup for Variable Temperature (VT) Powder Diffraction Studies

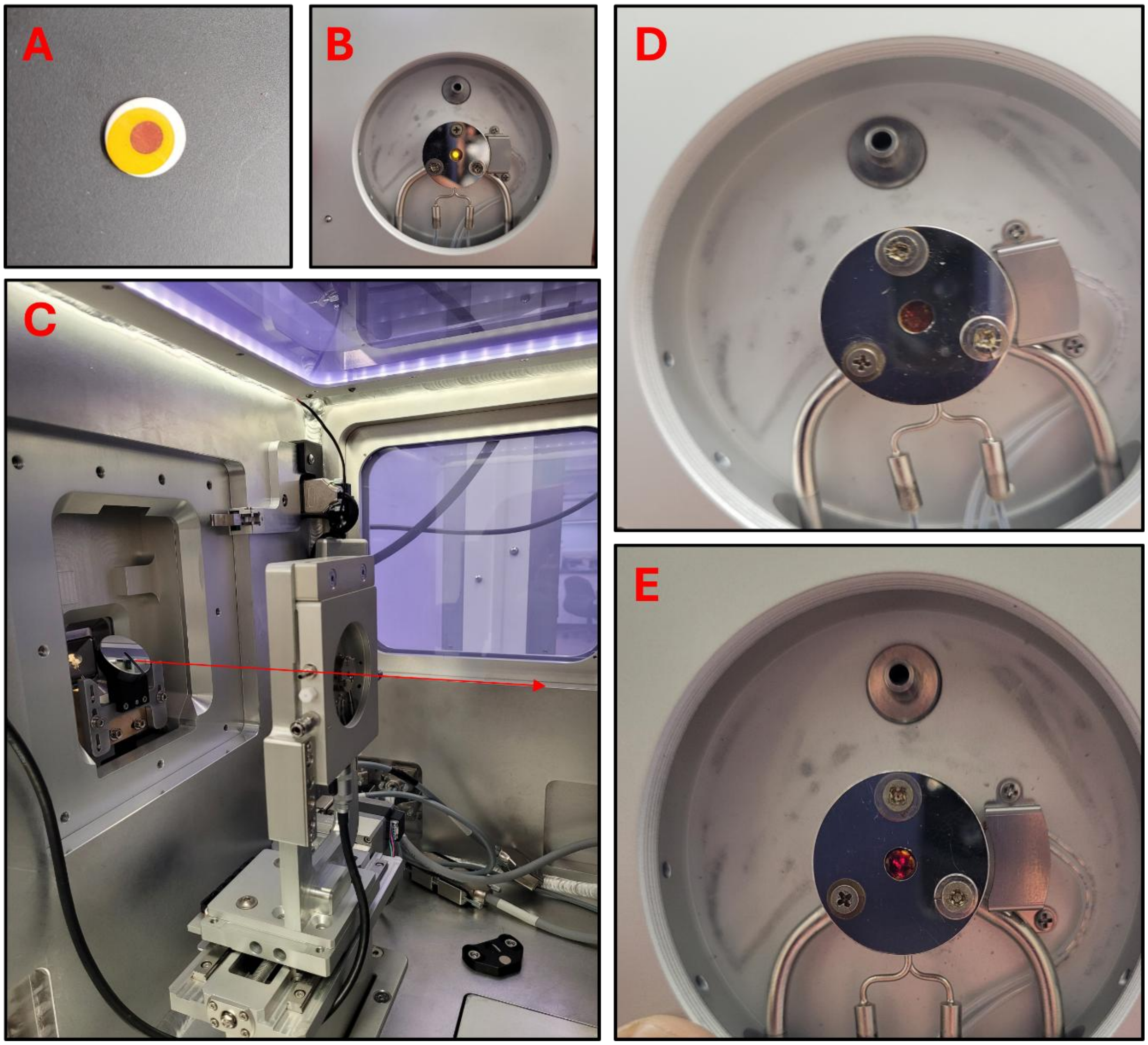

Figure S16. Images showing the setup of the powder diffraction experiment at variable temperatures. **Image A** shows the metallic washer packed with the polymer sample using Kapton tape on both sides. **Image B** shows the Linkam HFSX350 stage loaded with the blank sample (metallic washer with the Kapton tape on both sides). **Image C** shows the Linkam HFSX350 stage loaded on the instrument, the direction of the X-ray beam is shown with the red arrow. **Image D** shows the polymer sample on the Linkam HFSX350 stage before heating. **Image E** shows the polymer sample on the Linkam HFSX350 stage after heating.

Powder Diffraction Patterns

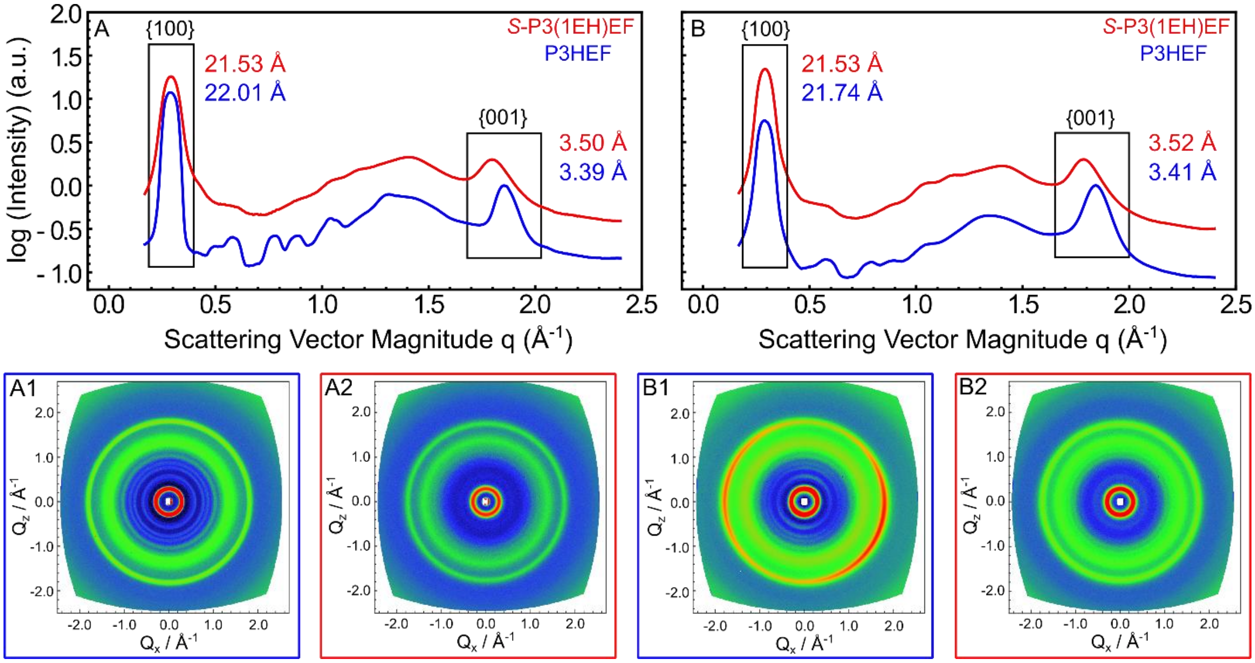

Figure S17. Top – Overlay of azimuthally averaged powder diffraction patterns of P3HEF (M_n = 8.2 kg/mol, blue) and *S*-P3(1EH)EF (M_n = 14.9 kg/mol, red), obtained from powdered polymer samples prepared from THF (A) and $CHCl_3$ (B) solutions at room temperature. The data in panel A are identical to those shown in the main manuscript and are included here to facilitate direct comparison with samples prepared from $CHCl_3$. **Bottom -** Corresponding 2D diffraction images of powdered samples from THF (A1, A2) and $CHCl_3$ (B1, B2) solutions at room temperature, confirming similar solid-state structural features in both solvent systems. While THF and $CHCl_3$ induce different behaviors in solution, no significant differences are observed in the solid-state packing, supporting the same structural conclusions regardless of the solvent used to prepare the polymer solutions.

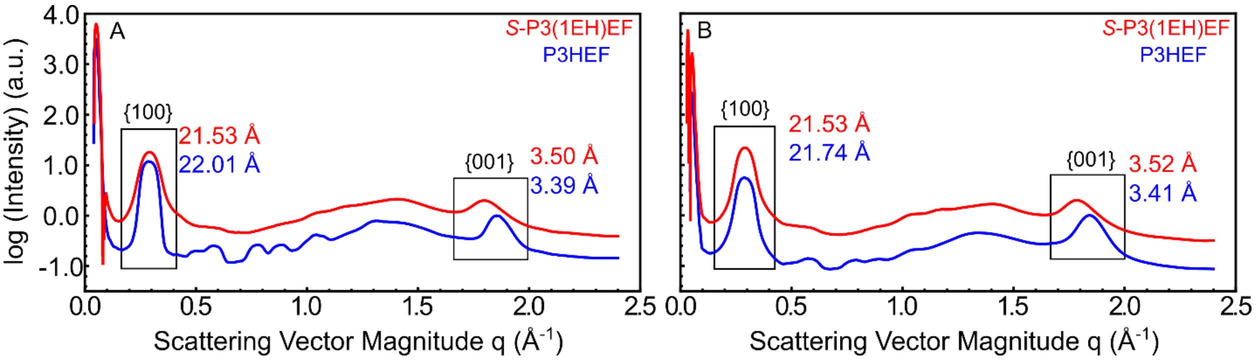

Figure S18. Full q-range version of the azimuthally averaged diffraction profiles shown in Figure S17. This includes the beam center region. The low-q region was omitted in Figure S17 for better visualization of high-q features, which appear less sharp when plotted on a full scale.

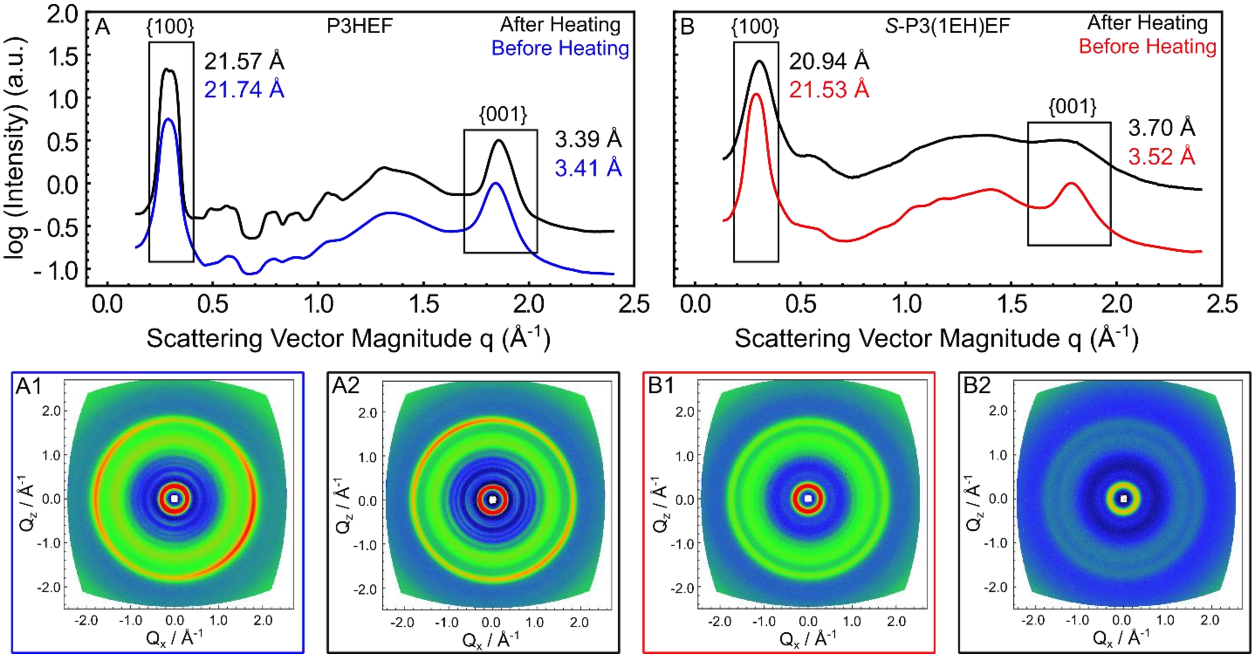

Figure S19. Top – Overlay of an azimuthally averaged powder diffraction pattern of P3HEF (M_n = 8.2 kg/mol, left), and *S*-P3(1EH)EF (M_n = 14.9 kg/mol, right) before and after heating using Linkam HFSX350. **Bottom** - Corresponding 2D images of the diffraction pattern for the powder sample before and after heating P3HEF (A1 and A2) and *S*-P3(1EH)EF (B1 and B2).

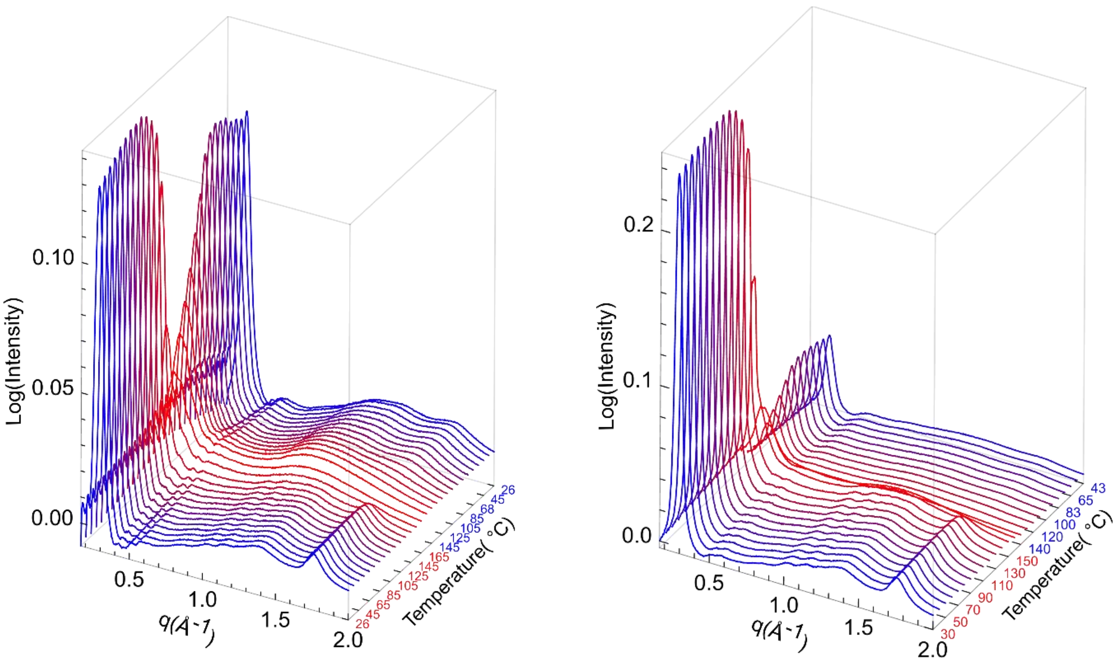

Figure S20. VT-powder X-ray scattering patterns for different runs of *S*-P3(1EH)EF (M_n = 14.9 kg/mol, $Đ$ = 1.36).

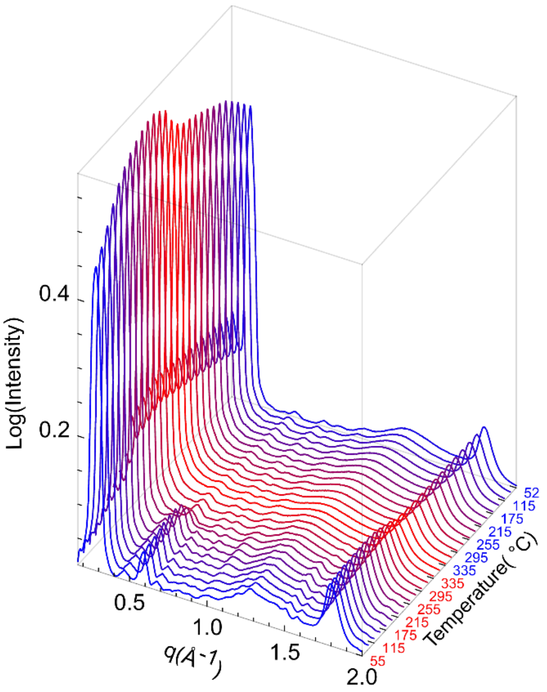

Figure S21. VT-powder X-ray scattering patterns of P3HEF (M_n = 8.2 kg/mol, $Đ$ = 1.28).

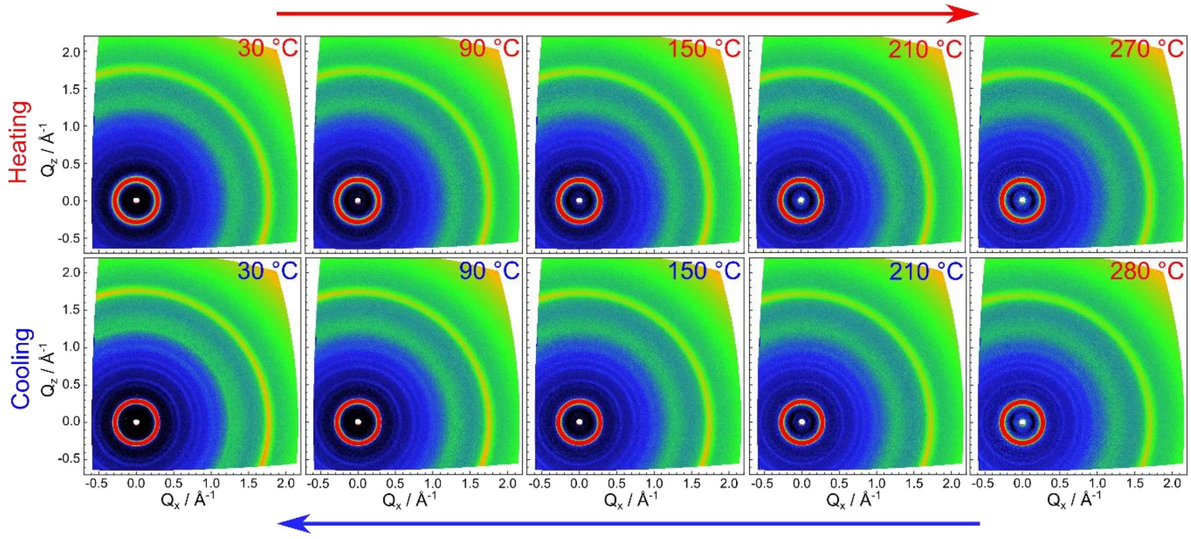

Figure S22. 2D images of the diffraction pattern for the powder sample of P3HEF (M_n = 8.2 kg/mol) at varying temperatures during heating using Linkam HFSX350 and uncontrolled cooling.

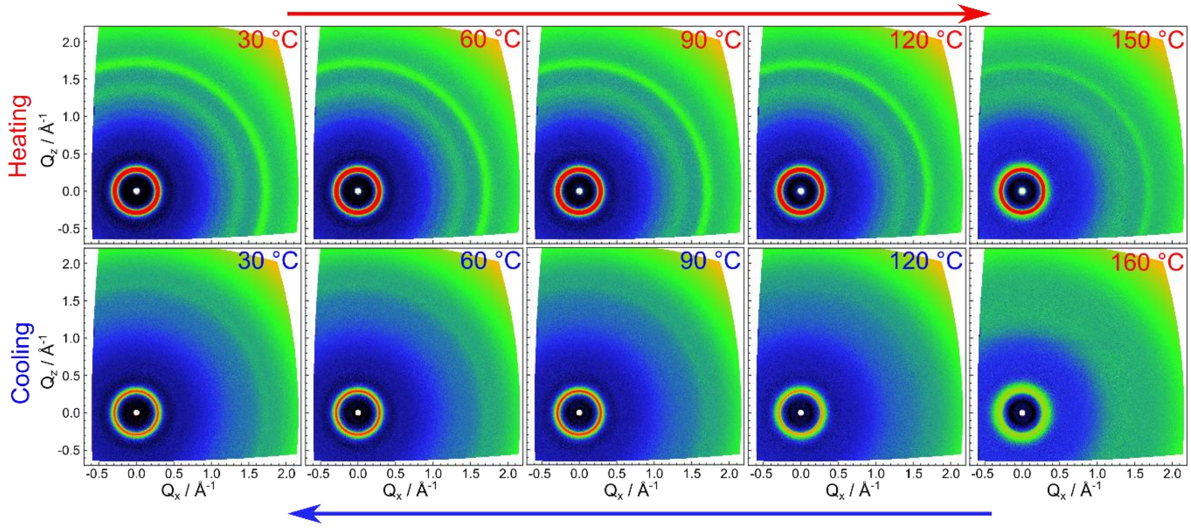

Figure S23. 2D images of the diffraction pattern for the powder sample of *S*-P3(1EH)EF (M_n = 14.9 kg/mol) at varying temperatures during heating using Linkam HFSX350 and uncontrolled cooling.

GIWAXS analysis of thin films cast on glass slides.

Note: Glass slides were used as substrates for casting films for solid-state CD measurements. Since silicon wafers were used in the GIWAXS studies reported in the manuscript, additional GIWAXS analysis was performed to confirm that the change in substrate did not affect polymer orientation. No significant differences were observed as isotropic scattering patterns were noted for *S*-P3(1EH)EF.

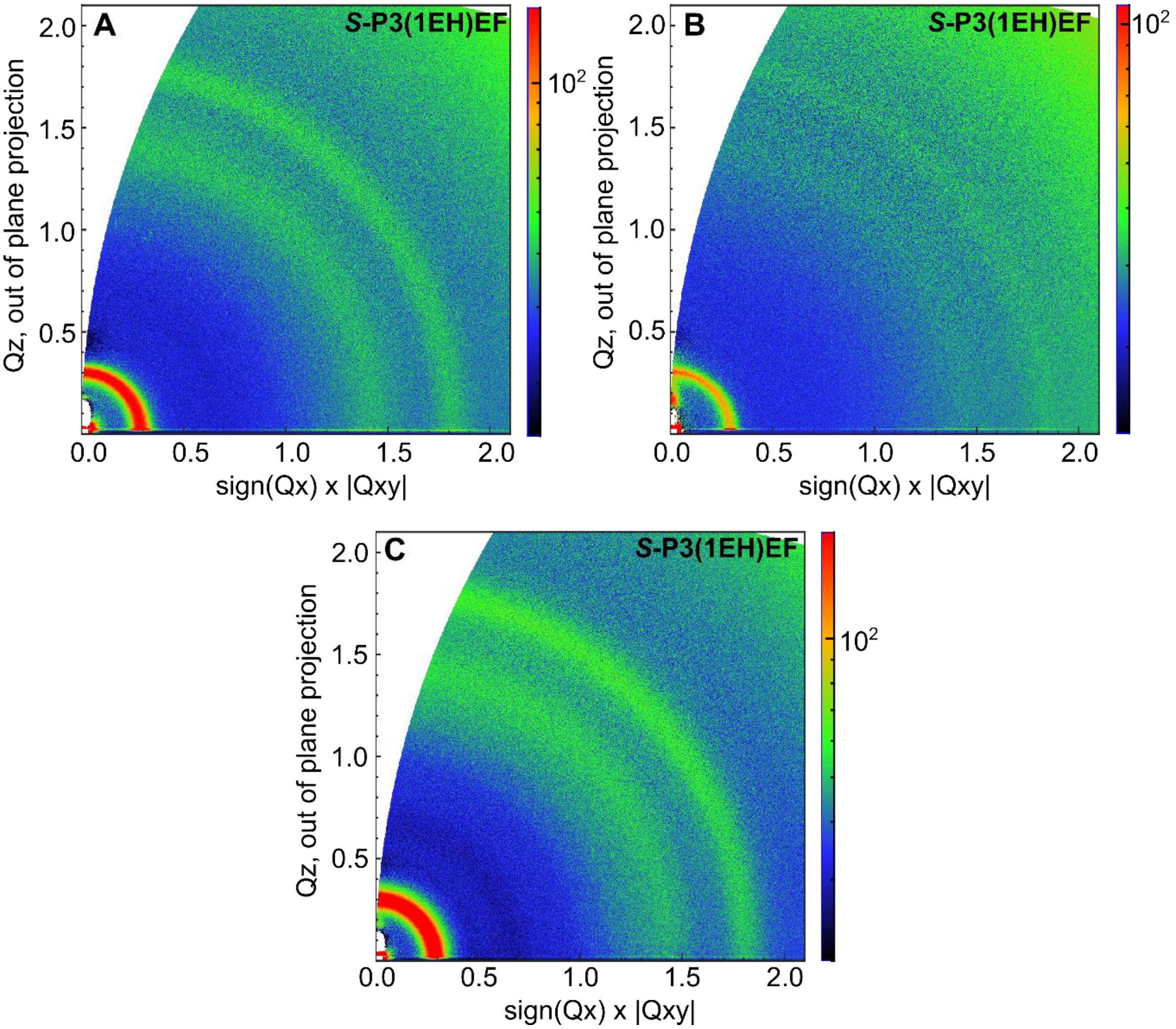

Figure S24. GIWAXS 2D images of *S*-P3(1EH)EF (M_n = 19.4 kg/mol) thin films cast on microscope glass slides (10 mm × 30 mm). Images **A** and **B** illustrate the GIWAXS pattern of thin films of *S*-P3(1EH)EF cast via solvent-vapor annealing procedure using THF and $CHCl_3$ as casting solvents, respectively. Additionally, image **C** presents the GIWAXS pattern of *S*-P3(1EH)EF cast via a drop-casting procedure using $CHCl_3$ as the casting solvent.

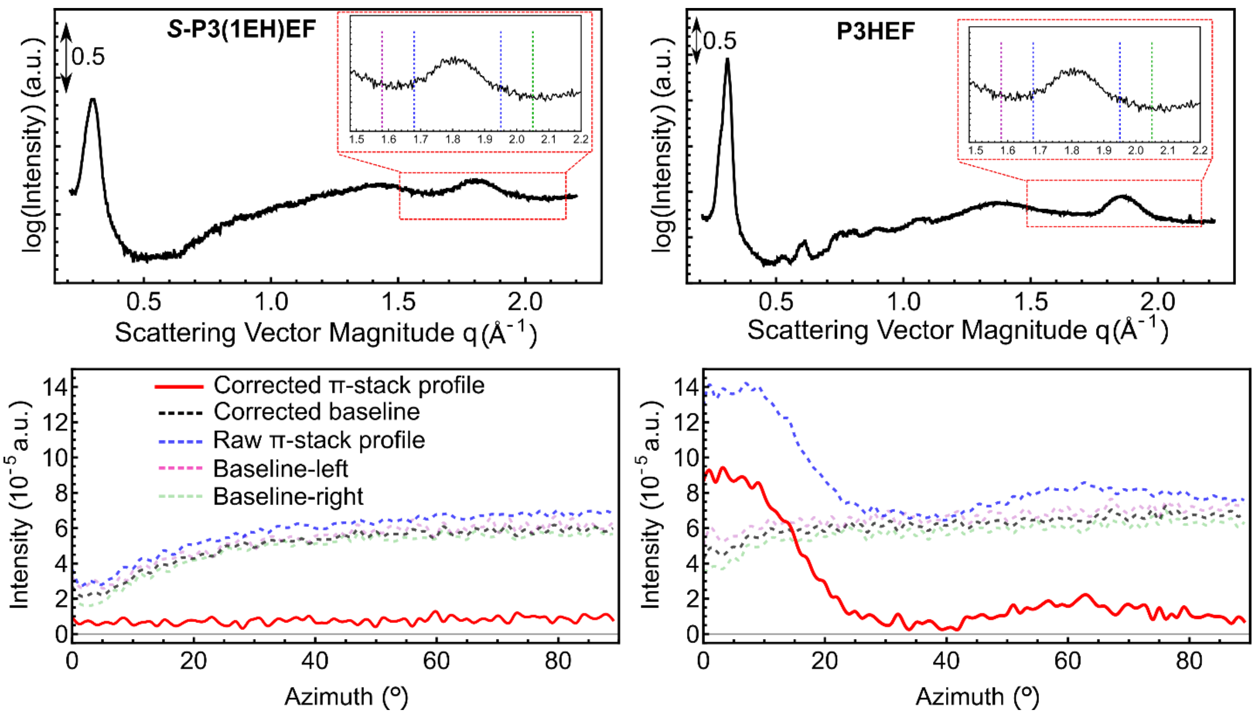

Figure S25. Azimuthal intensity profiles extracted from the GIWAXS patterns of *S*-P3(1EH)EF (left) and P3HEF (right). For *S*-P3(1EH)EF, the (001) π–π stacking peak is isotropically distributed. In contrast, P3HEF exhibits a predominantly edge-on orientation, with ~70% of the population integrated from 0–35°, and a minor off-axis component indicating an intermediate orientation between face-on and edge-on relative to the substrate.

Thermogravimetric Analysis (TGA)

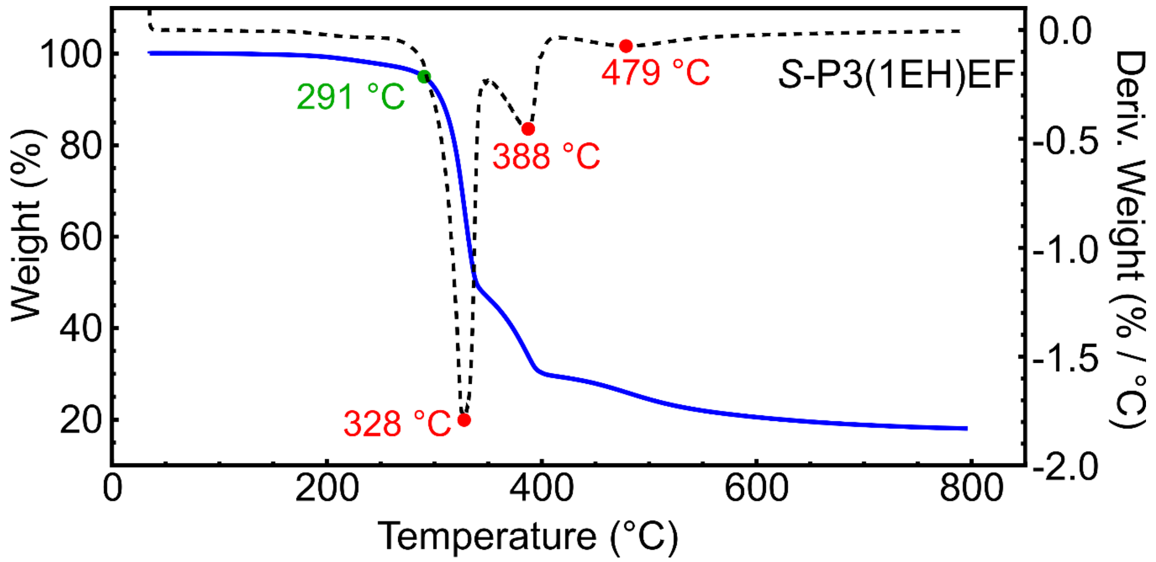

Figure S26. TGA curve of *S*-P3(1EH)EF (M_n = 17.3 kg/mol) showing the (derivative) weight loss as a function of temperature. $T_{d\ 5\%}$ = 291 °C, shown in green color, the first and second degradation transition temperatures are shown in red color.

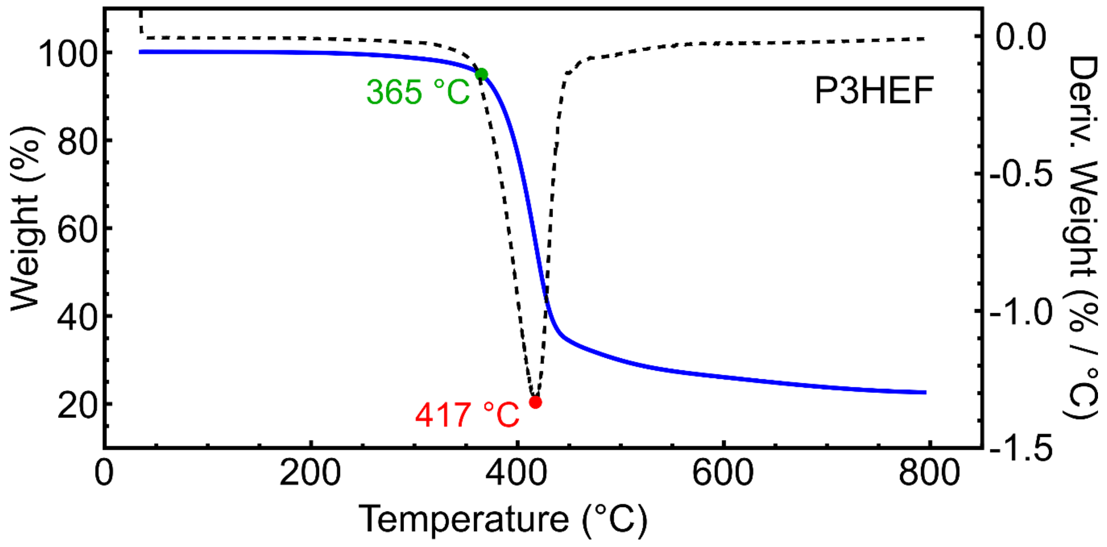

Figure S27. TGA curve of P3HEF (M_n = 10.2 kg/mol) showing the (derivative) weight loss as a function of temperature. $T_{d\ 5\%}$ = 365 °C, shown in green color, and the degradation transition temperature is shown in red color.

Differential Scanning Calorimetry (DSC) Analysis

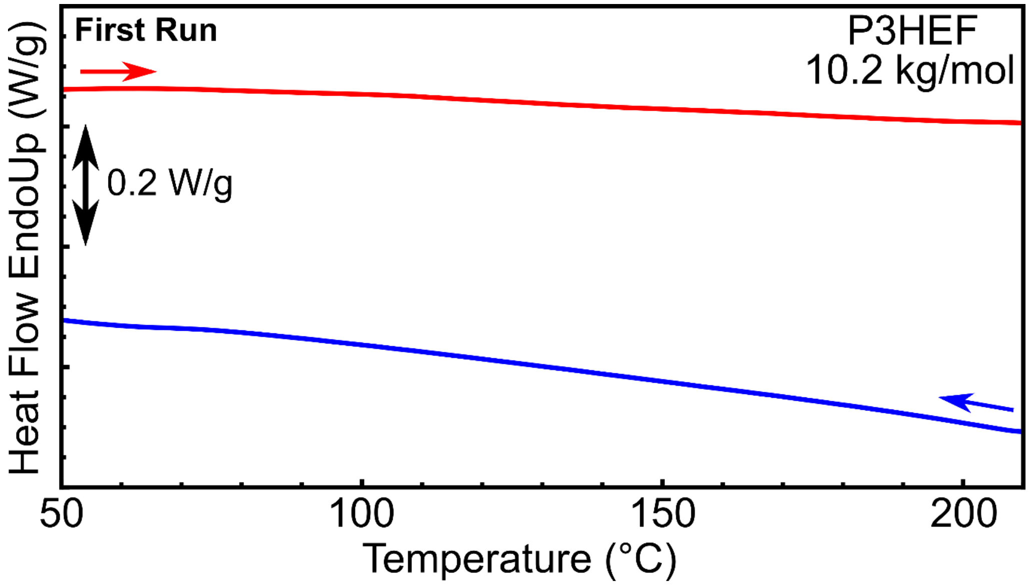

Figure S28. DSC curves (first run) of P3HEF (**M_n = 10.2 kg/mol**) measured at a rate of 10 °C/min.

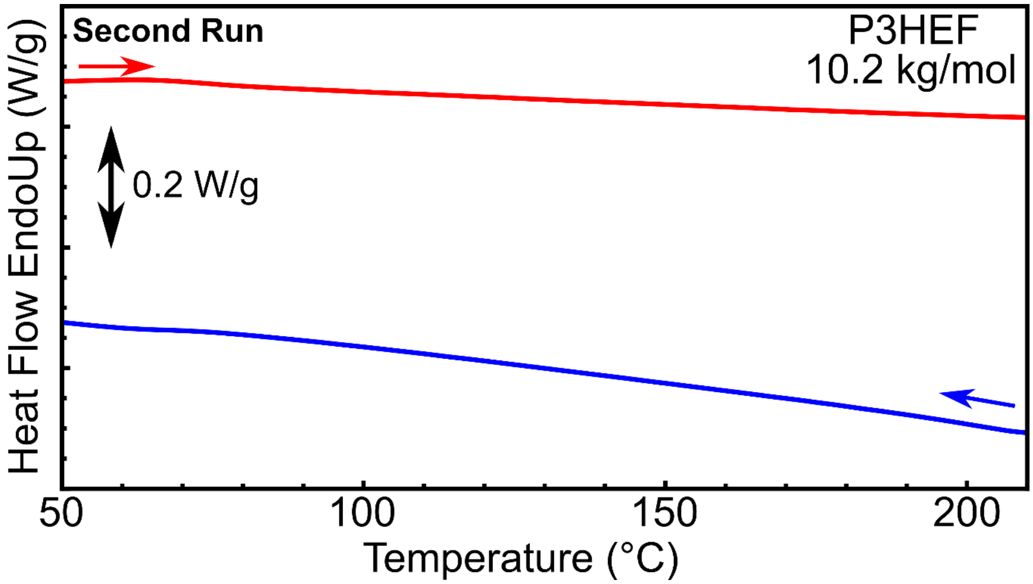

Figure S29. DSC curves (second run) of P3HEF (**M_n = 10.2 kg/mol**) measured at a rate of 10 °C/min.

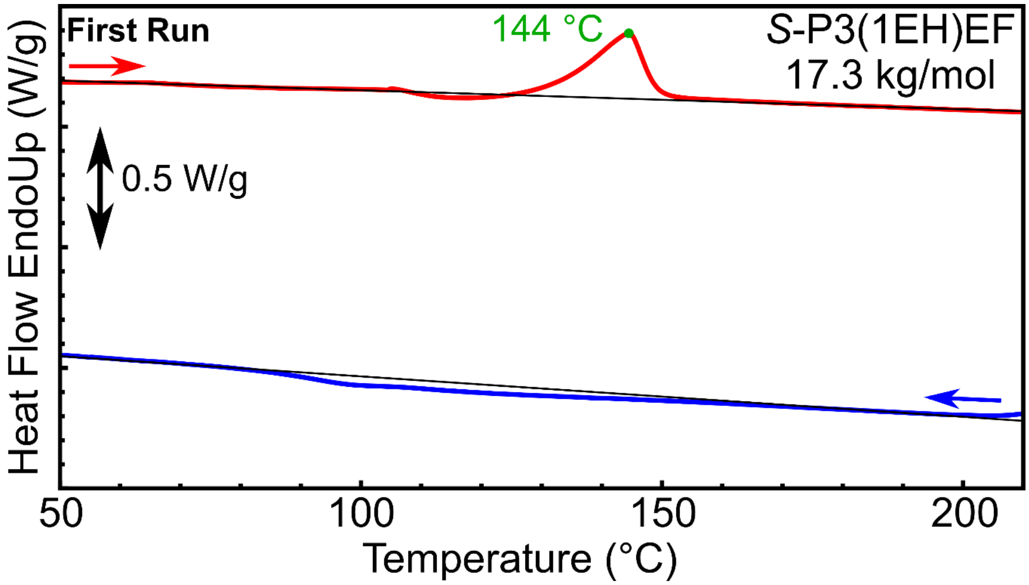

Figure S30. DSC curves (**first run**) of *S*-P3(1EH)EF (**M_n = 17.3 kg/mol**), measured at a rate of 10 °C/min.

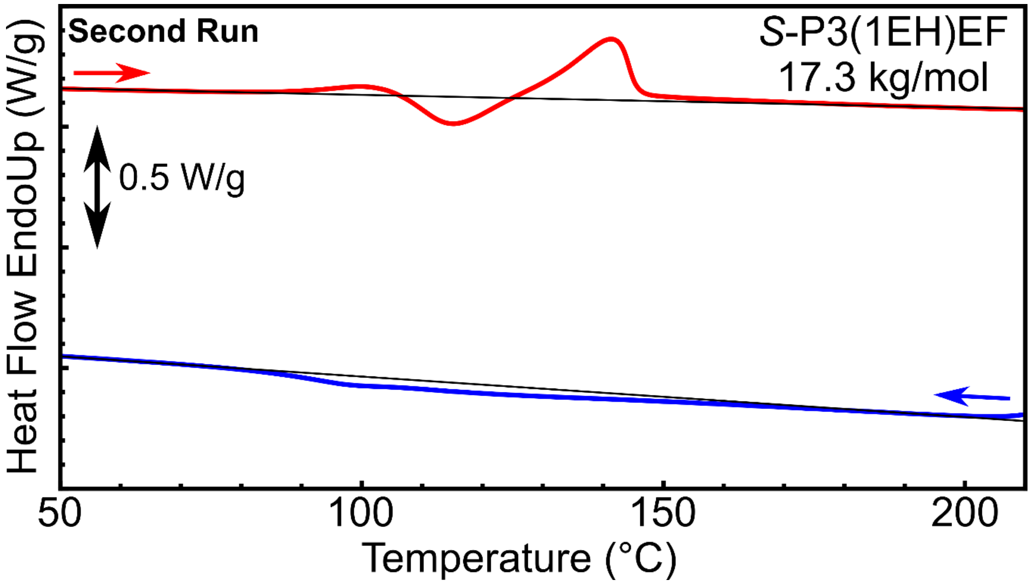

Figure S31. DSC curves (**second run**) of *S*-P3(1EH)EF (**M_n = 17.3 kg/mol**), measured at a rate of 10 °C/min.

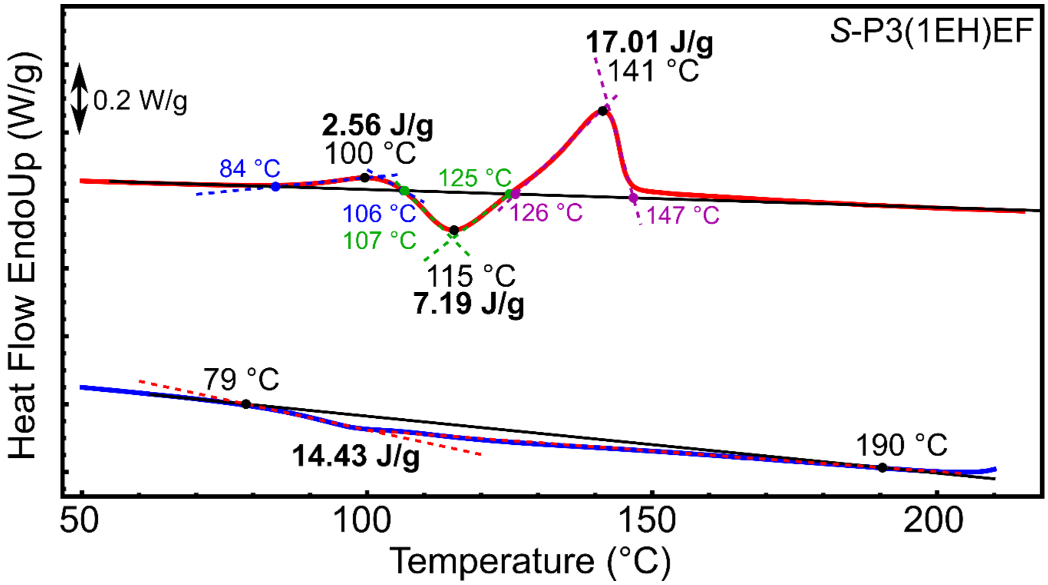

Figure S32. DSC curves (top: second heating run, and bottom: second cooling run) of *S*-P3(1EH)EF with M_n = 17.3 kg/mol, measured at a rate of 10 °C/min. The thermogram highlights the enthalpy of crystallization (ΔH_c) and enthalpy of fusion (ΔH_m) for each transition in bold black. Onset, endset, melting, and crystallization temperatures of the transitions are labeled in black.

Note: The *S*-P3(1EH)EF sample (17.3 kg/mol) undergoes a crystallization transition at 115 °C, just before its melting transition at 141 °C. This behavior is likely due to **cold crystallization** or post-crystallization of the polymer. This crystallization event is more pronounced in the second heating cycle than in the first.

Predicted CD and Absorbance Spectra for the P3MEF 13-mer.

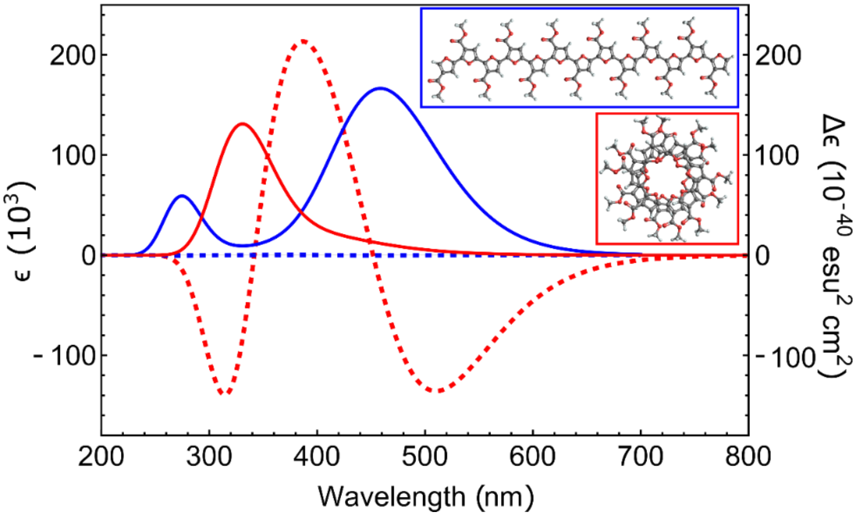

Figure S33. The predicted absorption and CD spectra (TDDFT CAM/B3LYP 6-31G(d,p)) for methylfuran-3-carboxylate (13-mers) with all furans either *anti* (blue) or *syn* (red). The helical furan has an *M* configuration (anticlockwise).

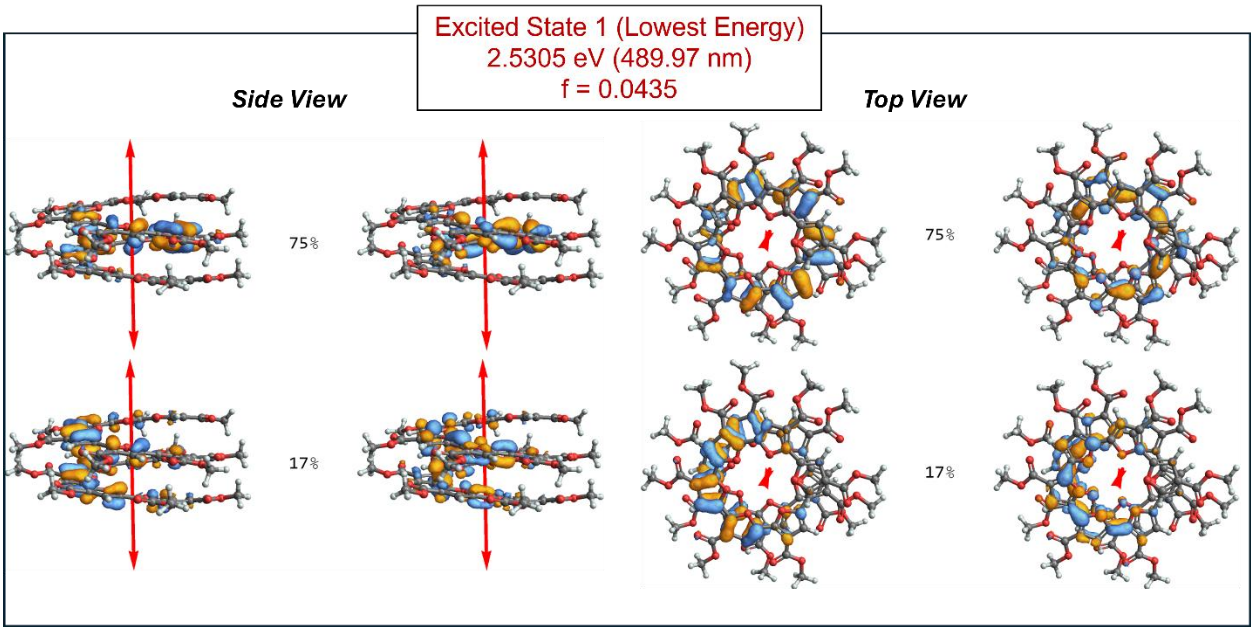

Figure S34. Natural transition orbital (NTO) representations for Excited State 1 (2.5305 eV, 489.97 nm; f = 0.0435) for all *syn* conformation of the methylfuran-3-carboxylate (13-mers). Side and top views are shown with red arrows indicating the orientation of the transition dipole moment.

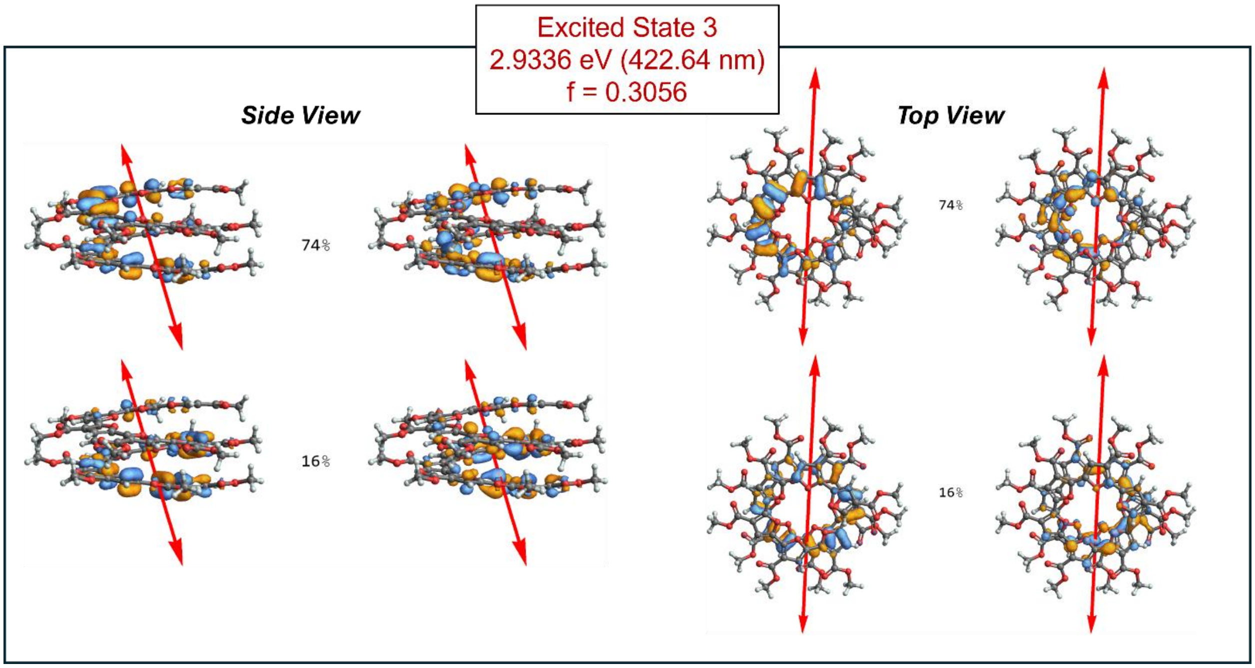

Figure S35. Natural transition orbital (NTO) representations for Excited State 3 (2.9336 eV, 422.64 nm; f = 0.3056) for all *syn* conformation of the methylfuran-3-carboxylate (13-mers). Side and top views are shown with red arrows indicating the orientation of the transition dipole moment.

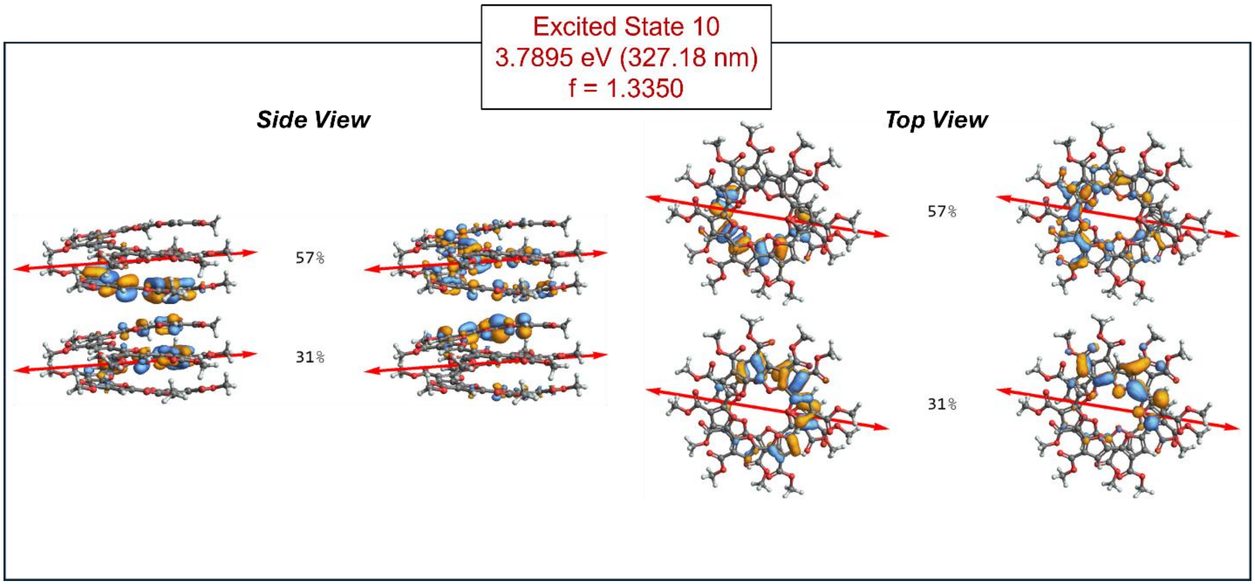

Figure S36. Natural transition orbital (NTO) representations for Excited State 10 (3.7895 eV, 327.18 nm; f = 1.3350) for all *syn* conformation of the methylfuran-3-carboxylate (13-mers). Side and top views are shown with red arrows indicating the orientation of the transition dipole moment.

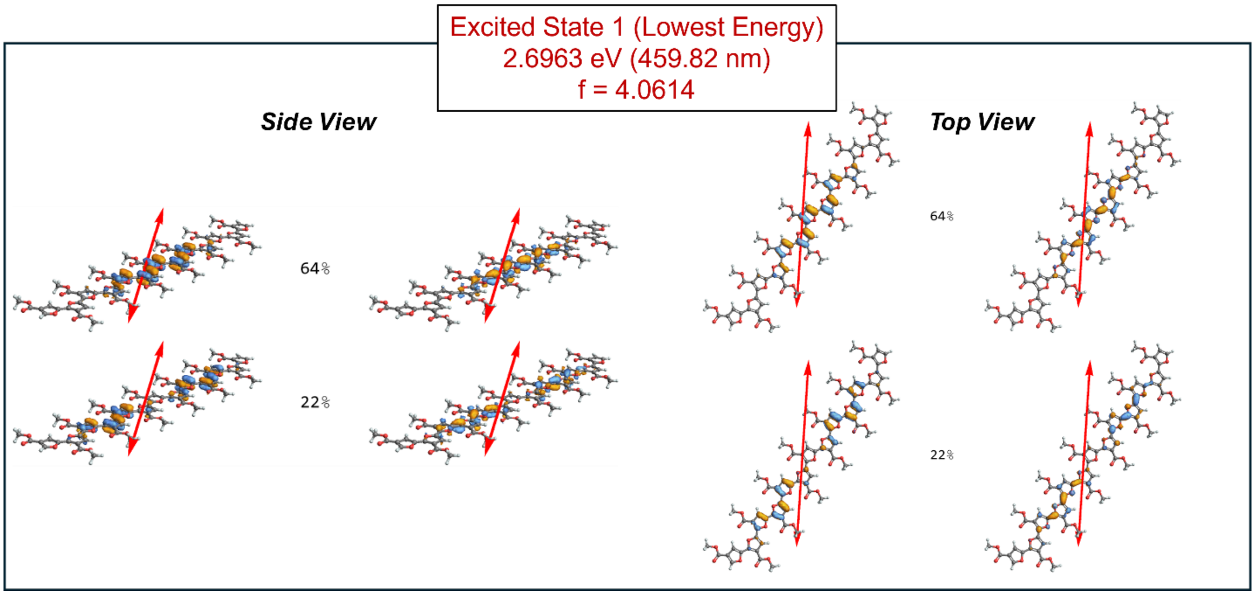

Figure S37. Natural transition orbital (NTO) representations for Excited State 1 (2.6936 eV, 459.82 nm; f = 4.0614) for all *anti* conformation of the methylfuran-3-carboxylate (13-mers). Side and top views are shown with red arrows indicating the orientation of the transition dipole moment.

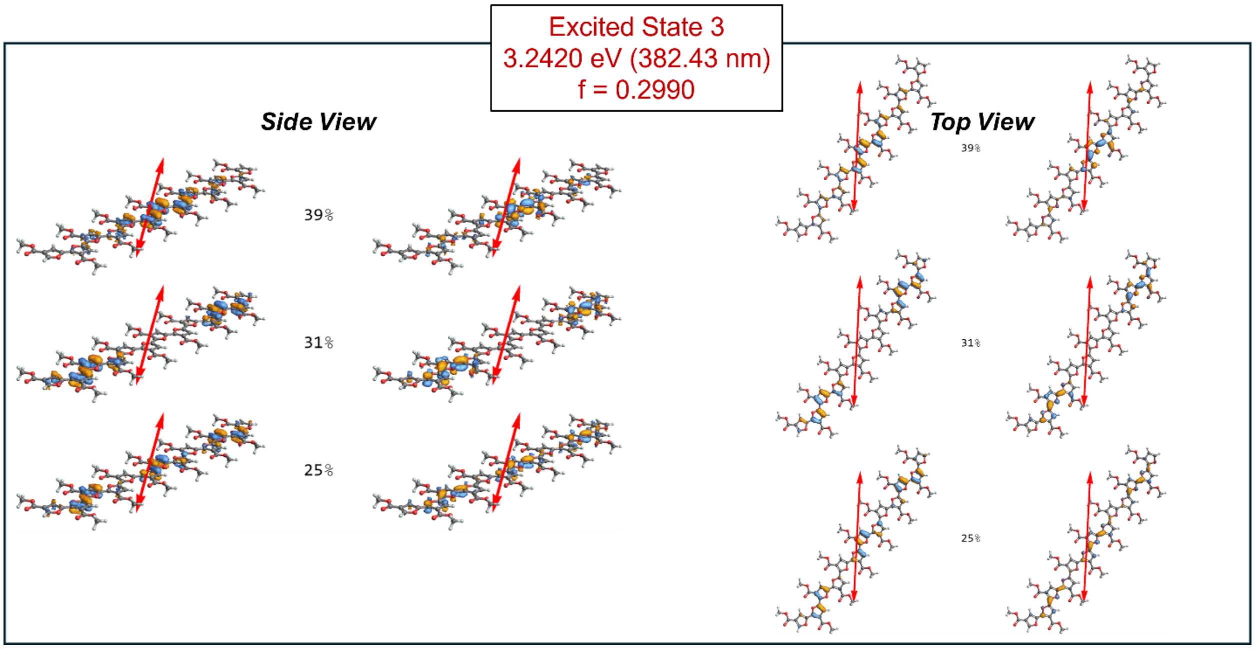

Figure S38. Natural transition orbital (NTO) representations for Excited State 3 (3.2420 eV, 382.43 nm; f = 0.2990) for all *anti* conformation of the methylfuran-3-carboxylate (13-mers). Side and top views are shown with red arrows indicating the orientation of the transition dipole moment.

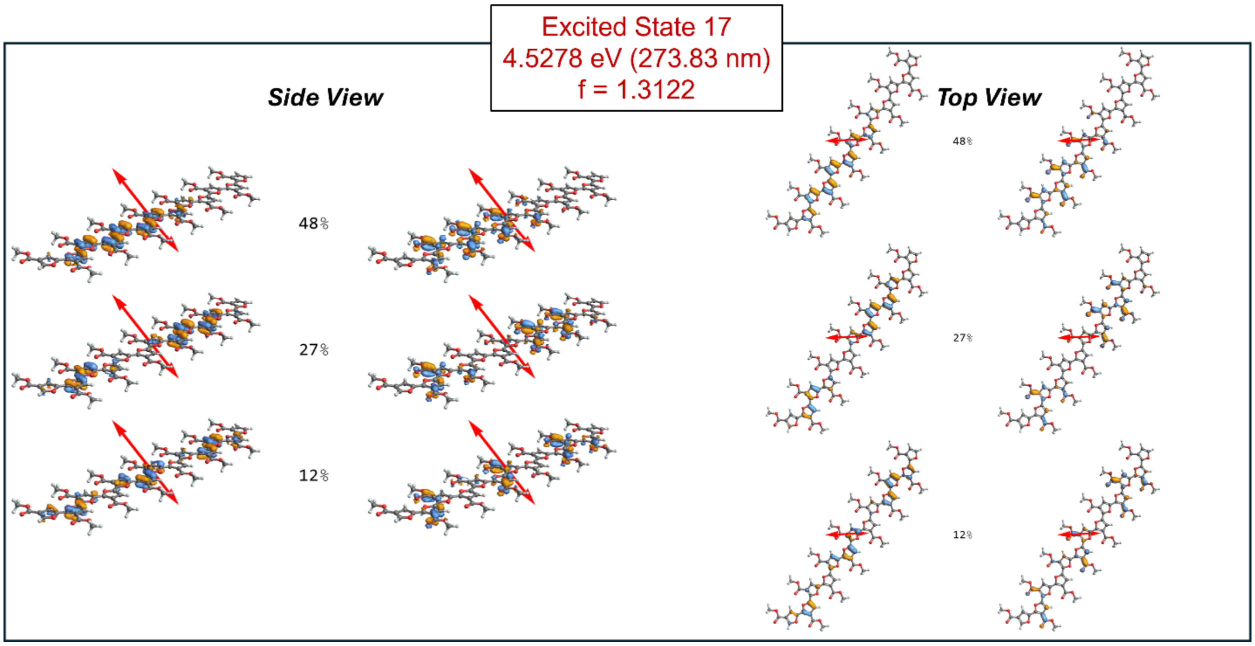

Figure S39. Natural transition orbital (NTO) representations for Excited State 17 (4.5278 eV, 273.83 nm; f = 1.3122) for all *anti* conformation of the methylfuran-3-carboxylate (13-mers). Side and top views are shown with red arrows indicating the orientation of the transition dipole moment.

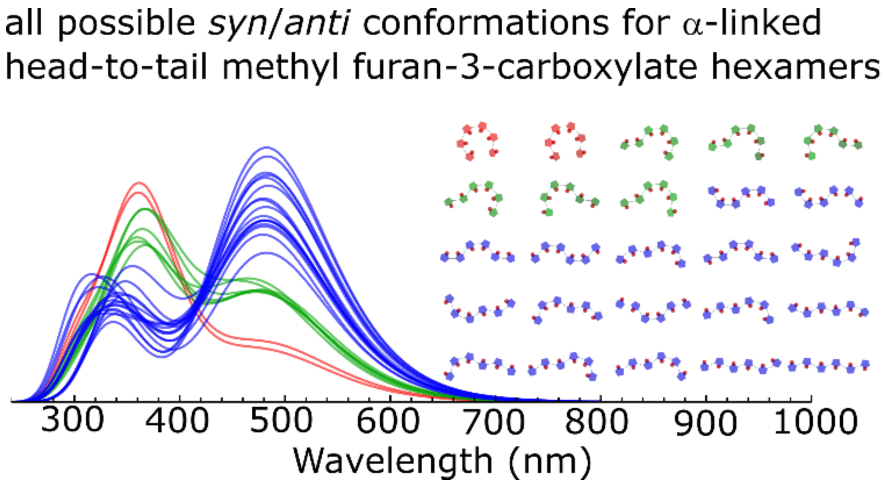

Figure S40. The predicted absorption spectra (TDDFT CAM-B3LYP 6-31G(d,p)) for methylfuran-3-carboxylate (6-mers) with varying proportions of *syn* and *anti* linkages.

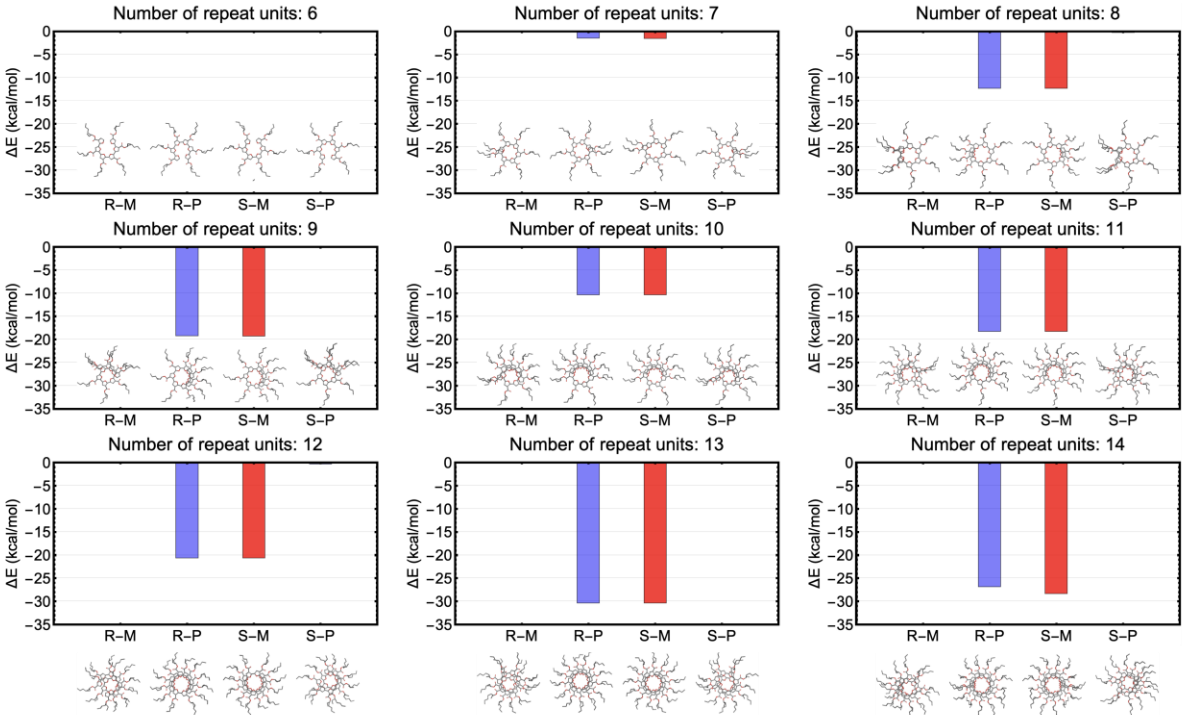

Figure S41. Helix-sense bias vs. oligomer length for 1-ethylhexyl ester side chain. Bar graphs show the relative energy difference ΔE (kcal mol^{-1}) between matched (*R–P, S–M*) and mismatched (*R–M, S–P*) screw-sense/stereocenter pairings in dendronized helices bearing ethyl–hexyl side chains, as a function of repeat units (n = 6–14). Structures were fully optimized at the GFN2-xTB level.

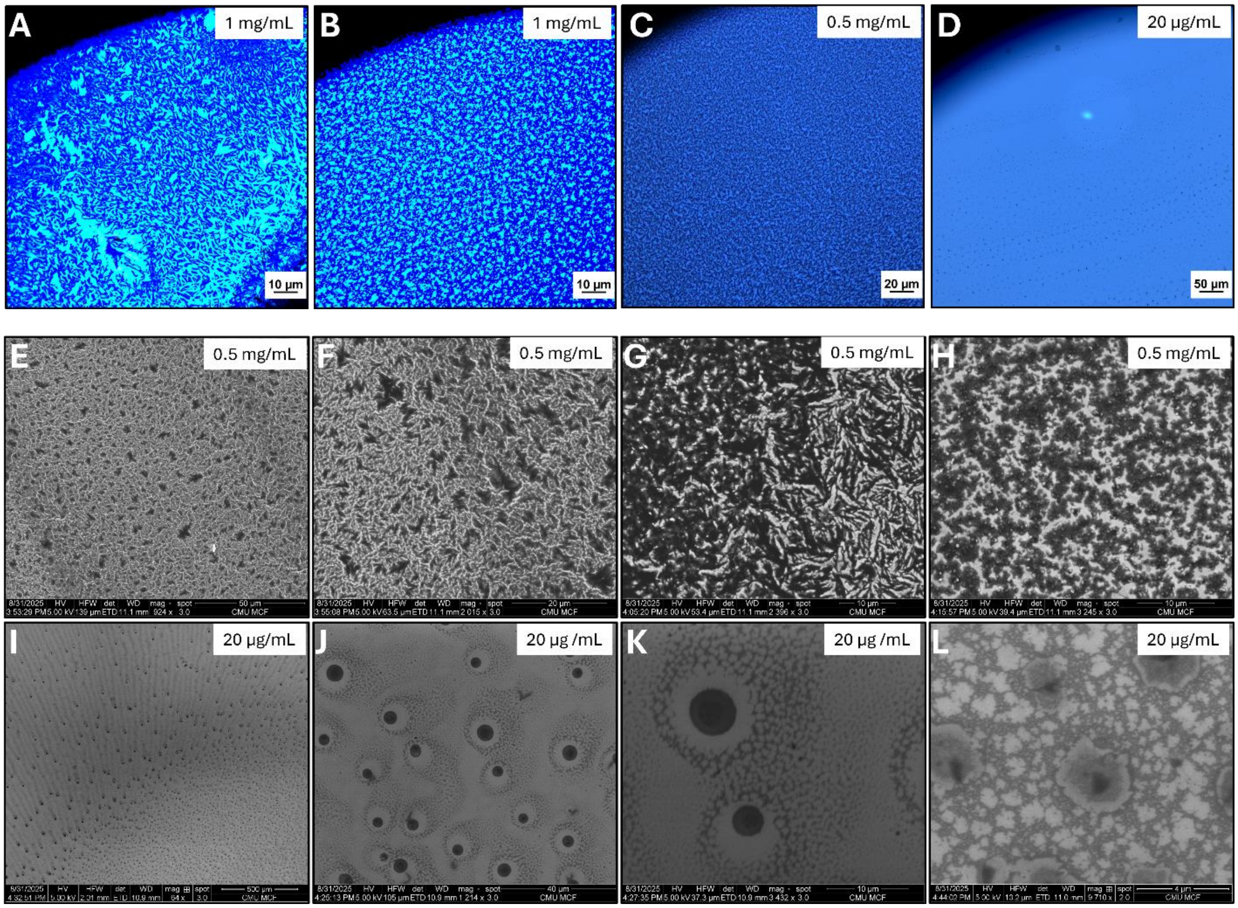

Figure S42. Representative optical microscopy (A–D) and SEM (E–L) images of P3HEF films prepared by drop-casting from THF solutions of varying concentration (1 mg/mL, 0.5 mg/mL, and 20 µg/mL) onto silicon wafers. Images were collected under identical conditions to allow direct comparison.

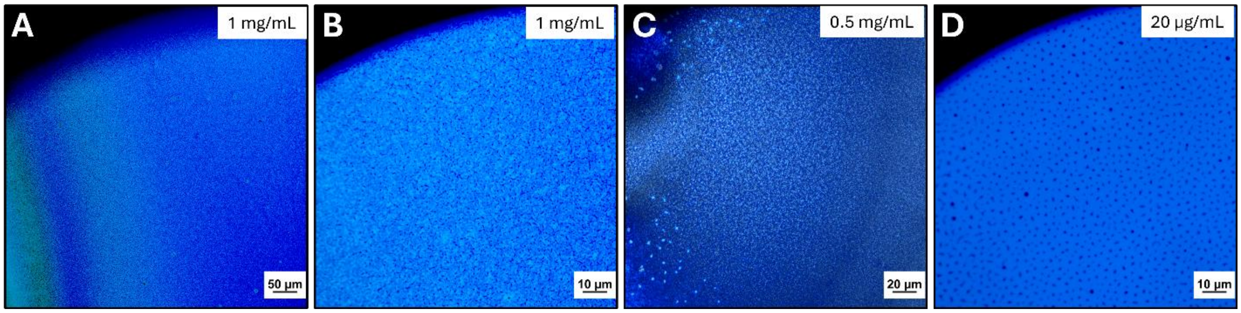

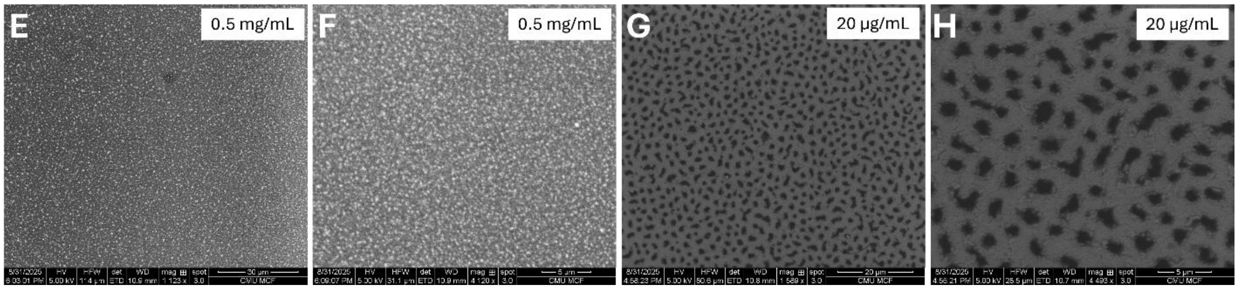

Figure S43. Representative optical microscopy (A–D) and SEM (E–H) images of P3HEF films prepared by drop-casting from $CHCl_3$ solutions of varying concentration (1 mg/mL, 0.5 mg/mL, and 20 µg/mL) onto silicon wafers. Images were collected under identical conditions to allow direct comparison.

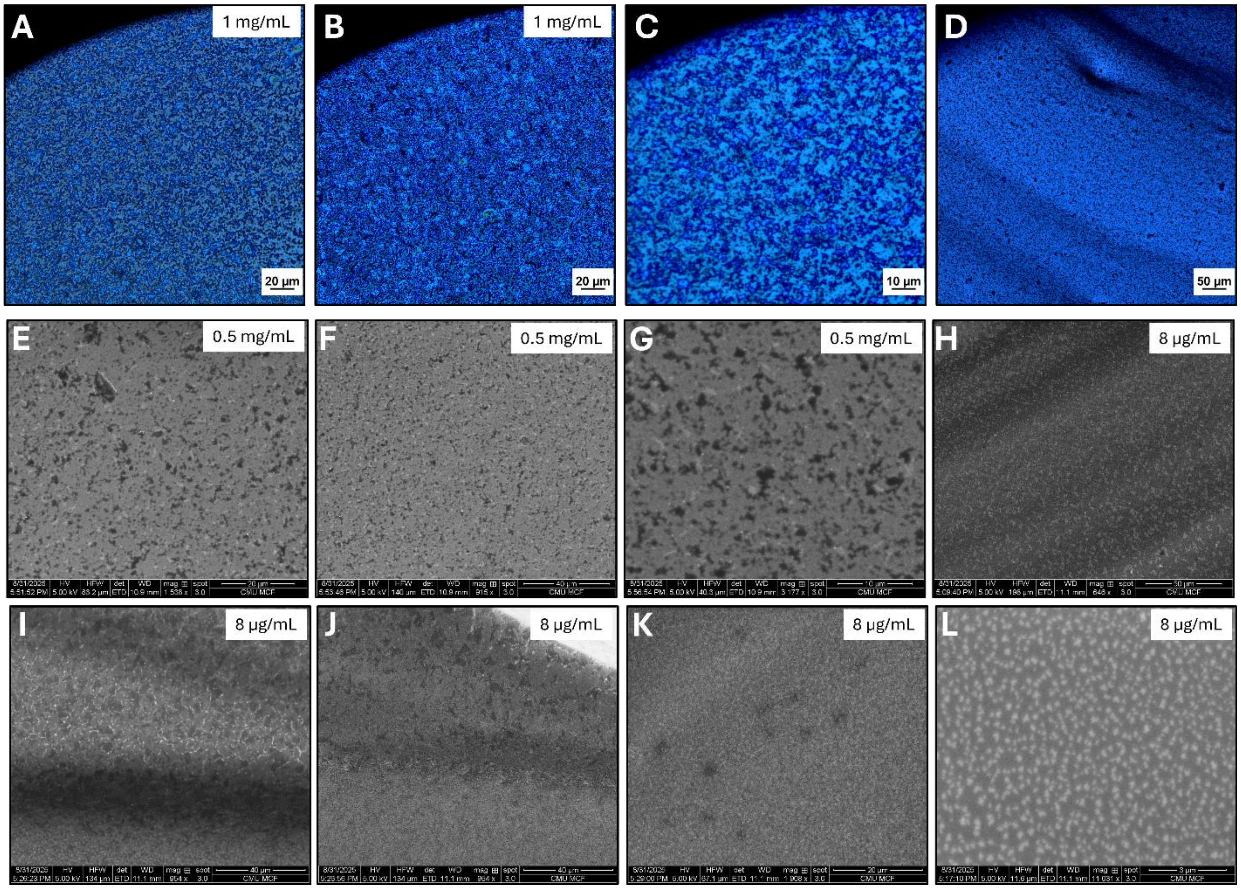

Figure S44. Representative optical microscopy (A–D) and SEM (E–L) images of *S*-P3(1EH)EF films prepared by drop-casting from THF solutions of varying concentration (1 mg/mL, 0.5 mg/mL, and 8 µg/mL) onto silicon wafers. Images were collected under identical conditions to allow direct comparison.

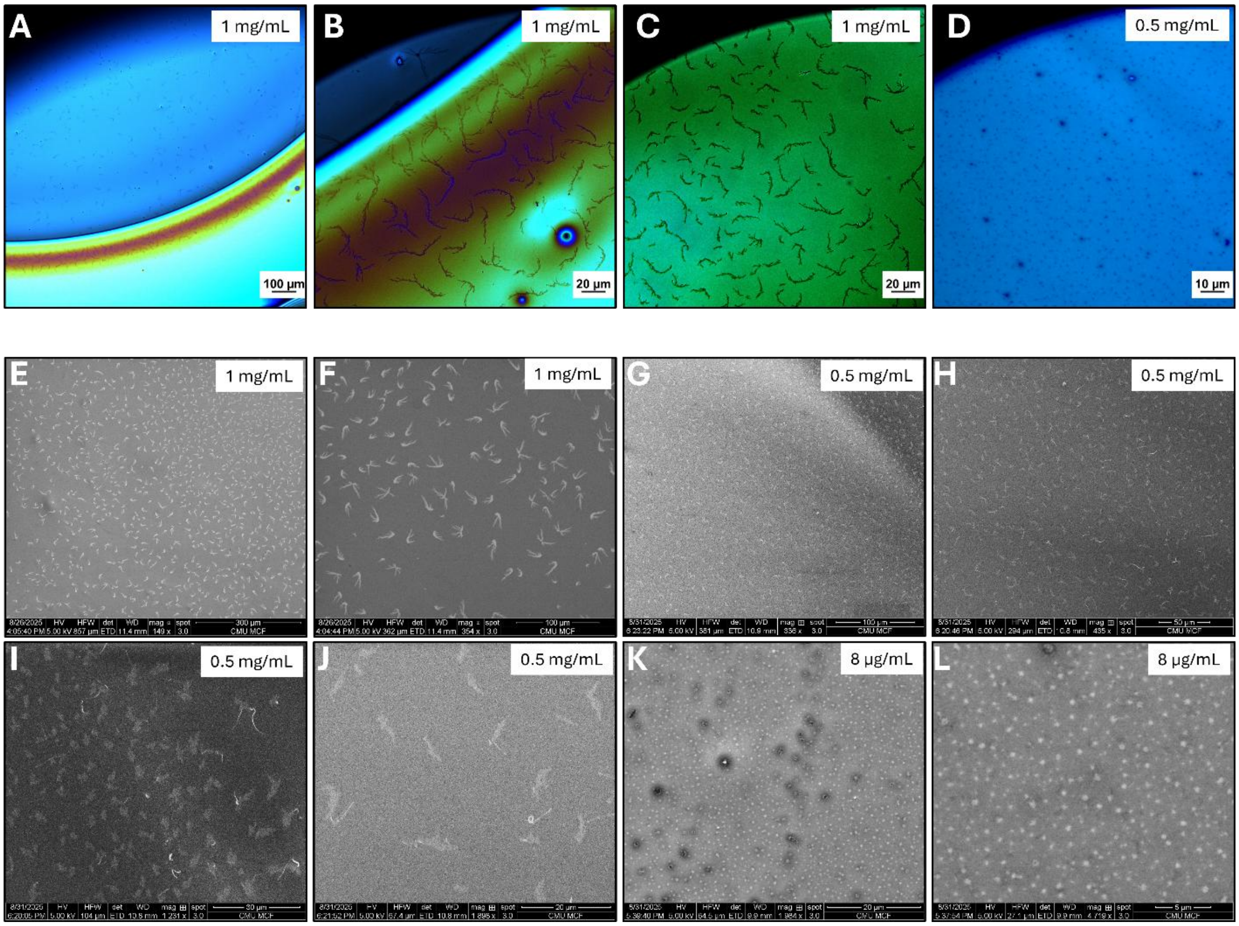

Figure S45. Representative optical microscopy (A–D) and SEM (E–L) images of *S*-P3(1EH)EF films prepared by drop-casting from $CHCl_3$ solutions of varying concentration (1 mg/mL, 0.5 mg/mL, and 8 µg/mL) onto silicon wafers. Images were collected under identical conditions to allow direct comparison.

References

(1) Varni, A. J.; Fortney, A.; Baker, M. A.; Worch, J. C.; Qin, Y. Y.; Yaron, D.; Bernhard, S.; Noonan, K. J. T.; Kowalewski, T. Photostable Helical Polyfurans. *J. Am. Chem. Soc.* **2019**, *141*, 8858-8867.

(2) Kawakami, M.; Schulz, K. H. G.; Varni, A. J.; Tormena, C. F.; Gil, R. R.; Noonan, K. J. T. Statistical copolymers of thiophene-3-carboxylates and selenophene-3-carboxylates; 77Se NMR as a tool to examine copolymer sequence in selenophene-based conjugated polymers. *Polym. Chem.* **2022**, *13*, 5316-5324.
